# Gaussian curvature–driven direction of cell fate toward osteogenesis with triply periodic minimal surface scaffolds

**DOI:** 10.1073/pnas.2206684119

**Published:** 2022-10-03

**Authors:** Yuhe Yang, Tianpeng Xu, Ho-Pan Bei, Lei Zhang, Chak-Yin Tang, Ming Zhang, Chenjie Xu, Liming Bian, Kelvin Wai-Kwok Yeung, Jerry Ying Hsi Fuh, Xin Zhao

**Affiliations:** ^a^Department of Biomedical Engineering, The Hong Kong Polytechnic University, Hung Hom, Hong Kong SAR, China;; ^b^Department of Mechanical and Aerospace Engineering, Hong Kong University of Science and Technology, Kowloon, Hong Kong SAR, China;; ^c^Department of Industrial and Systems Engineering, The Hong Kong Polytechnic University, Hung Hom, Kowloon, Hong Kong SAR, China;; ^d^Department of Biomedical Engineering, City University of Hong Kong, Kowloon, Hong Kong SAR, China;; ^e^School of Biomedical Sciences and Engineering, South China University of Technology, Guangzhou 510006, China;; ^f^National Engineering Research Center for Tissue Restoration and Reconstruction, South China University of Technology, Guangzhou 510006, China;; ^g^Department of Orthopaedics & Traumatology, Li Ka Shing Faculty of Medicine, The University of Hong Kong, Pokfulam, Hong Kong SAR, China;; ^h^Department of Mechanical Engineering, National University of Singapore, 117575, Singapore

**Keywords:** TPMS, hyperboloidal structure, bone regeneration, mesenchymal stem cells

## Abstract

Leaf photosynthesis, coral mineralization, and trabecular bone growth depend on triply periodic minimal surfaces (TPMSs) with hyperboloidal structure on every surface point with varying Gaussian curvatures. However, translation of this structure into tissue-engineered bone grafts is challenging. This article reports the design and fabrication of high-resolution three-dimensional TPMS scaffolds embodying biomimicking hyperboloidal topography with different Gaussian curvatures, composed of body inherent β-tricalcium phosphate, by stereolithography-based three-dimensional printing and sintering. The TPMS bone scaffolds show high porosity and interconnectivity. Notably, compared with conventional scaffolds, they can reduce stress concentration, leading to increased mechanical strength. They are also found to support the attachment, proliferation, osteogenic differentiation, and angiogenic paracrine function of human mesenchymal stem cells (hMSCs). Through transcriptomic analysis, we theorize that the hyperboloid structure induces cytoskeleton reorganization of hMSCs, expressing elongated morphology on the convex direction and strengthening the cytoskeletal contraction. The clinical therapeutic efficacy of the TPMS scaffolds assessed by rabbit femur defect and mouse subcutaneous implantation models demonstrate that the TPMS scaffolds augment new bone formation and neovascularization. In comparison with conventional scaffolds, our TPMS scaffolds successfully guide the cell fate toward osteogenesis through cell-level directional curvatures and demonstrate drastic yet quantifiable improvements in bone regeneration.

## Significance

This article reports an exciting breakthrough in the translation of the biomimicking hyperboloidal structure into three-dimensional tissue-engineered bone grafts and reveals how the nature-derived hyperboloid structure affects osteogenesis and angiogenesis for expedited bone regeneration. We fabricated triply periodic minimal surfaces (TPMSs) embodied with hyperboloidal topography with different Gaussian curvatures. The wavy TPMS scaffolds directed the osteogenic differentiation and angiogenic paracrine of mesenchymal stem cells through the hyperboloidal topography-induced cytoskeleton reorganization and nuclear deformation, accelerating bone regeneration. We believe that these features can grant our scaffolds application as simple, safe, efficient, and personalized bone grafts with notable clinical translation potential.

Hyperboloid is a common topological structure in nature (1, 2). For example, coral, a living fossil dating up to 500 million years ago, maintains a hyperboloidal feature on its mineralized surface (3). Coincidentally, plant leaves, a product of natural selection after millions of years, also develop such hyperboloidal structures (4). Hyperboloidal structure, with a positive and a negative curvature in two perpendicular directions at one point, is deemed beneficial for promoting coral calcification and increasing photosynthesis by enlarging the exposed surface area of the leaves (5, 6). Intriguingly, the microstructure of mammalian trabecular bone also demonstrates such hyperboloid structure (7–9). Hyperboloid structures could be the universal result of evolution directed optimization, yet their potential role in bone development has not been touched upon.

Recently, it has been recognized that the surface topological structure (e.g., concave or convex) has significant influence on regulating stem cell behaviors and functions and regenerating bone tissues (10–16). For example, a convex surface may promote osteogenesis of mesenchymal stem cells (MSCs) since the cytoskeletal force on the convex surface deforms the cell nucleus and increases the lamin A levels (10). The substrate topography could also affect the protein aggregation on the MSC membrane and subsequently activate different cell signaling pathways (e.g., Rho, Wnt, FAK, TGF-β/BMP) that manipulate cell differentiation (17–20). For instance, the mean curvature of curved surfaces could modulate the cell contractility to determine the shape and growth behavior of osteoid-like tissues (21). We thus hypothesize that the hyperboloidal structure could modulate the behaviors and functions of stem cells involved in bone regeneration. However, how or why such hyperboloidal structure affects stem cell behaviors and functions and bone regeneration remains unknown.

**Fig. 1.**
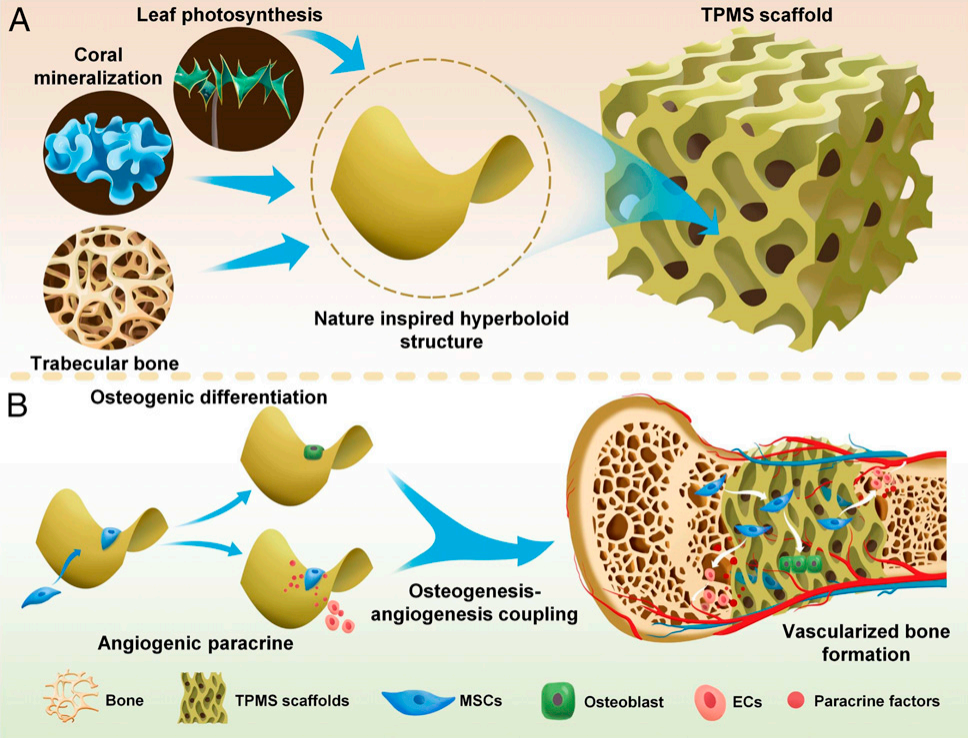
Schematics showing the design and application of biomimicking 3D TPMS scaffolds. (*A*) TPMS scaffolds with hyperboloidal topology are inspired by coral mineralization, leaf photosynthesis, and trabecular bone microstructure. (*B*) TPMS scaffolds with hyperboloidal surfaces can regulate the behaviors and functions of mesenchymal stem cells (MSCs) in terms of osteogenic differentiation and angiogenic paracrine for osteogenesis–angiogenesis coupling and vascularized bone regeneration. EC, endothelial cell.

Here, we designed triply periodic minimal surface (TPMS) structured three-dimensional (3D) scaffolds that embody biomimicking hyperboloidal topography with varying Gaussian curvatures (Fig. 1*A*). TPMS is a series of infinite, non-self-intersecting periodic surface structures in three principal directions (2, 22). The TPMS has a hyperboloidal structure on every surface point, with varying Gaussian curvatures. Gaussian curvature *Κ* of a surface at a point is the product of the principal curvatures, *K*_1_ (positive curvature, a convex surface) and *K*_2_ (negative curvature, a concave surface) (23, 24). The scaffolds are fabricated with body inherent β-tricalcium phosphate (β-TCP) by stereolithography-based 3D printing and sintering. We performed a thorough optimization of printing and sintering parameters, achieving control over the 3D scaffold structures with excellent resolution, accuracy, and reproducibility. The resultant 3D TPMS scaffolds have high porosity, excellent interconnectivity, and impressive mechanical property (smoothly curved surfaces to eliminate stress concentration). The hyperboloidal topography enables the adhesion, proliferation, osteogenic differentiation, and angiogenic paracrine of human mesenchymal stem cells (hMSCs). Cells on the hyperbolic surfaces show contracted cell shape in the concave (*K*_2_ < 0) direction, whereas they present snail-like configuration in the convex (*K*_1_ > 0) direction (Fig. 1*B*). Such curvature-induced cytoskeleton reorganization results in cytoskeletal contractility and nucleus deformation with higher lamin A/C expression, leading to osteogenesis–angiogenesis coupling, which is critical for accelerated bone regeneration. Furthermore, the rabbit femoral defect model demonstrates the impressive performance of our TPMS scaffolds in terms of new bone formation. The mouse subcutaneous implantation model further validates the scaffolds’ substantial potential in supporting tissue infiltration and neovascularization. We envision that these well-defined features can grant our TPMS scaffolds application as a safer and more efficient bone graft with notable clinical translation potential. Our design will also provide guidelines to design simple, efficient, and personalized bone grafts with simultaneous osteogenesis and angiogenesis; the TPMS concept is also transferable to designing other bone implants such as metal or polymer prostheses.

## Results and Discussion

### Fabrication and Characterization of 3D TPMS Scaffolds.

**Fig. 2.**
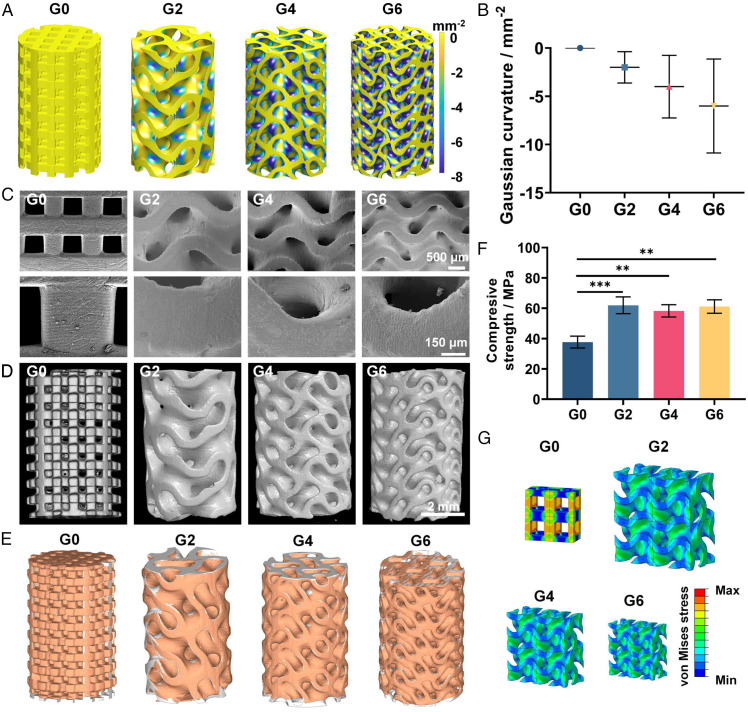
Characterization of the 3D TPMS scaffolds. (*A*) Gaussian curvature distribution and (*B*) quantification of Gaussian curvature of the designed scaffolds. (*C*) SEM images of the 3D-printed TPMS scaffolds. (*D*) Representative reconstructed micro-CT images of the TPMS scaffolds. (*E*) Geometric completeness of the scaffolds assessed by comparing a computer-aided design model (gray) with the corresponding 3D shapes (orange) reconstructed by micro-CT. (*F*) Compressive strength of TPMS scaffolds and (*G*) von Mises stress distribution of the conventional truss scaffold (G0) and TPMS scaffolds under compression with four unit cells analyzed; G0, G2, G4, and G6 mean Gaussian curvature distribution of 0, −2, −4 and −6 mm^−2^, respectively. Sample size *n* = 3 for all experiments by one-way ANOVA with Tukey’s post hoc test for multiple comparisons. Data are presented as mean ± SD. **P < 0.01 and ***P < 0.001 denote statistical significance.

We first designed the gyroid-type TPMS scaffolds with different average Gaussian curvatures of –2, –4, and –6 mm^−2^ (denoted as G2, G4, and G6) with 60% porosity (Fig. 2*A*). Gyroid-type was chosen because it has excellent fluid permeability and mechanical properties (25). Gaussian curvatures of –2, –4, and –6 mm^−2^ and 60% porosity were chosen as they were in range of natural trabecular bone (8). The conventional truss scaffold with 60% porosity and 0 Gaussian curvature was used as control (denoted as G0). In geometric modeling, the curvature mapping was calculated for all scaffolds in which the G2, G4, and G6 groups presented distinct Gaussian curvature distribution from –2 to –6 mm^−2^, while the G0 group showed 0 Gaussian curvature on all surfaces of the scaffolds (Fig. 2 *A* and *B*). We then fabricated β-TCP TPMS scaffolds by using stereolithography-based 3D printing. We used FDA-approved β-TCP ceramic [Ca_3_(PO_4_)_2_] due to its excellent biocompatibility (26). We mixed the nano-sized β-TCP powder with photo-crosslinkable resin to make the 3D printing bone ink, then printed the 3D TPMS scaffolds by stereolithography-based 3D printing and further reinforced them by sintering. We went through a thorough optimization of printing and sintering parameters, achieving control over the 3D scaffold structures with excellent resolution, accuracy, and reproducibility. Representative scanning electron microscope (SEM) images of the fabricated scaffolds presented smooth and dense surfaces with no visible defects and excellent resolution of the printing process, with an accurate recapitulation of the hyperboloidal topology (Fig. 2*C*). The spatially internal structure of the scaffolds was evaluated by high-resolution micro–computed tomography (micro-CT) analysis (Fig. 2*D*). All scaffolds presented excellent interconnected pores with ∼60% porosity, which is beneficial for neotissue infiltration and nutrient exchange (*SI Appendix*, Fig. S1*A*) (27, 28). Moreover, with the increased Gaussian curvature of the TPMS scaffolds, we found there was an increase in the surface area (from 373 to 674 mm^2^) and decrease in pore size (from 0.9756 to 0.5632 mm) (*SI Appendix*, Fig. S1 *B* and *C*). Additionally, the geometric completeness of the scaffolds was assessed by comparing its computer-aided design model (gray) with the corresponding 3D shape (orange) reconstructed by micro-CT (Fig. 2*E*). All scaffolds presented high overlap in the whole 3D structure. Quantitatively, the reproducibility of porosity, mean pore size, and strut thickness of all printed scaffolds ranged from 93.33% to 98.89% (*SI Appendix*, Table S1), indicating the accurate printing process and representation of the topology characteristics of our designed scaffolds.

We then investigated the mechanical properties of the scaffolds with different Gaussian curvatures. We found that the TPMS groups presented compressive strength of 61.93 ± 5.54 MPa (G2), 58.27 ± 4.05 MPa (G4), and 61.13 ± 4.45 MPa (G6), respectively, which was significantly higher compared to the control group (G0) of 37.67 ± 3.92 MPa with the same porosity (Fig. 2*F*). This was because the TPMS design could effectively minimize the stress concentration and facilitate the load bearing capacity under compression (Fig. 2*G*). Notably, the compressive strength of the TPMS scaffolds is comparable to the mechanical property of natural trabecular bone (10–70 MPa) with the same porosity (29). In all, we successfully fabricated high-resolution 3D TPMS scaffolds with excellent reproducibility, biomimetic hyperboloidal topography, varying Gaussian curvatures, and appropriate mechanical properties.

### Cytocompatibility of TPMS Scaffolds.

On account of successful scaffold fabrication, we moved on to evaluate the cytocompatibility of the 3D TPMS scaffolds (*SI Appendix*, Fig. S2). We directly seeded hMSCs onto the scaffolds and found that the cells could adhere well onto both flat surfaces of the G0 group and hyperboloidal surfaces of the G2, G4, and G6 groups (*SI Appendix*, Fig. S2*A*). All groups presented over 95% cell viability as assessed by live/dead cell viability assay (*SI Appendix*, Fig. S2*B*). To further evaluate the cell seeding efficiency and cell density on different porous scaffolds, we dissociated the cells and calculated the cell density by normalizing the cell number by the surface area of the scaffolds. We found all scaffolds could support cell proliferation (*SI Appendix*, Fig. S2*C*). In addition, the cell density (cell number/surface area) on the hyperboloidal surfaces of the G2, G4, and G6 groups was significantly higher than that of the flat surface of the G0 group on days 1, 3, and 7. There was also no significant difference in cell density between TPMS groups (*SI Appendix*, Fig. S2*D*). This higher cell proliferation of the TPMS scaffolds could be attributed to the curved surfaces with increased cell retention rate and cell adhesion efficiency (30, 31). These results indicated the distinct cytocompatibility of the TPMS scaffolds.

### Osteogenesis of hMSCs on TPMS Scaffolds.

**Fig. 3.**
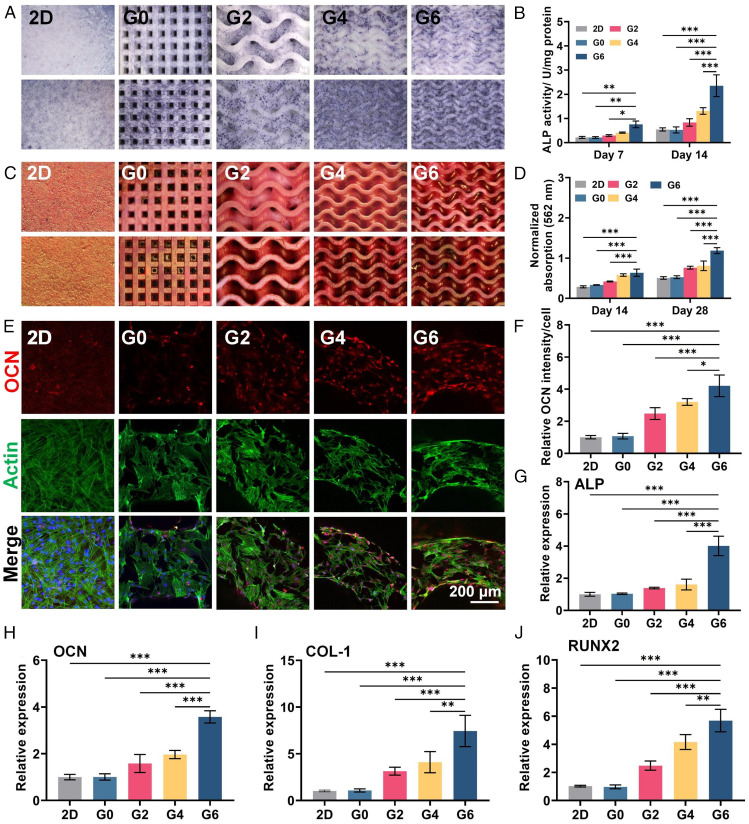
Osteogenesis evaluation of hMSCs seeded on different TPMS scaffolds. (*A*) ALP staining and (*B*) quantification of hMSCs on days 7 and 14. (*C*) ARS staining and (*D*) quantification of hMSCs on days 14 and 28. (*E* and *F*) Immunofluorescence staining and quantification of OCN of hMSCs seeded on different TPMS scaffolds after 7 d of incubation. (*G*–*J*) Relative expression of osteogenic-related gene expression including ALP, OCN, COL-1, and runt-related transcription factor 2 (RUNX2) of hMSCs on day 7. Sample size *n* = 3 for all experiments by one-way or two-way ANOVA with Tukey’s post hoc test for multiple comparisons. Data are presented as mean ± SD. *P < 0.05, **P < 0.01, and ***P < 0.001 denote statistical significance.

To assess the effect of the hyperboloidal structure on the osteogenic potential of hMSCs, we performed an in vitro osteogenesis evaluation by seeding hMSCs onto the TPMS scaffolds. Moreover, to exclude the possible effect of lower cell density on the osteogenesis evaluation of the G0 group as demonstrated above, we also adopted the 2D flat β-TCP plate (denoted as 2D), with the same cell density of TPMS scaffolds serving as a standard control. Alkaline phosphatase (ALP), which is an early marker of osteogenesis and plays an important role in osteoblastic differentiation, was first evaluated by staining and activity quantification (32). The three TPMS groups (G2, G4, and G6) presented more staining area compared to the G0 group and the control flat group (Fig. 3*A*). In addition, the G6 group demonstrated the largest staining area on both time points (days 7 and 14). Quantitatively, the G4 and G6 groups presented 2.46- and 4.46-fold greater ALP activity than the G0 group and 2.39- and 4.30-fold greater than that of the control flat group at day 14 (Fig. 3*B*). To evaluate the degree of extracellular matrix (ECM) mineralization of hMSCs on different TPMS scaffolds, alizarin red S (ARS) staining was conducted because this assay is a common strategy to evaluate the ECM mineralization in cells or tissues (33, 34). We first validated the feasibility of ARS staining to evaluate the tricalcium phosphate–based TPMS scaffolds. We used ARS to stain the pure scaffolds without any cells and found the red calcium nodule could be washed away with deionized water (*SI Appendix*, Fig. S3*A*). However, the scaffolds seeded with hMSCs presented obvious red calcium nodules after washing (*SI Appendix*, Fig. S3*B*). This result justified the use of ARS staining to evaluate the calcium nodule formation on the tricalcium phosphate–based scaffolds. The ARS staining results showed that after 14 d of incubation, more calcium nodule deposition was formed on the G2, G4, and G6 groups (Fig. 3*C*). The quantification analysis normalized by cell number revealed that the G6 group exhibited ∼1.91-fold and 2.25-fold greater mineralization compared to the G0 group after 14 and 28 d, respectively (Fig. 3*D*). The immunofluorescence staining of osteocalcin (OCN, osteogenic marker) also showed more positive staining on the TPMS groups than the G0 and control groups (Fig. 3 *E* and *F*). Moreover, analysis of the osteogenic gene expression of hMSCs supports the notion that the TPMS groups could promote the osteogenic differentiation of hMSCs with significantly increased expression of osteogenic genes including ALP, OCN, collagen-1 (COL-1) and runt-related transcription factor 2 (RUNX-2) (Fig. 3 *G*–*J*). Collectively, these results indicated that the TPMS scaffolds with hyperboloidal topology could effectively promote the hMSCs’ osteogenic differentiation compared to the G0 group and the flat control group with same cell density, and the G6 group with the largest Gaussian curvature showed the best promotion efficacy.

### Angiogenic Paracrine Response of hMSCs on TPMS Scaffolds.

As the paracrine effect of MSCs has proven capability to promote angiogenesis of endothelial cells to accelerate neovascularization for faster bone regeneration (35, 36), we further examined the potential role of the hyperboloidal topology on angiogenic paracrine response of hMSCs. To prepare the conditioned culture medium, hMSCs were seeded onto different TPMS scaffolds at a density of 1 × 10^4^ cells/cm^2^ and cultured in mixed medium (minimum essential medium α [α MEM]: endothelial cell medium [ECM] = 1:1) for 3 d. We then used the conditioned medium to culture human umbilical vein endothelial cells (HUVECs) (5 × 10^4^ cells/cm^2^) to investigate the angiogenic paracrine function of hMSCs. When the HUVECs became confluent, we applied the wound healing assay to evaluate the paracrine effect of hMSCs on HUVEC migration (*SI Appendix*, Fig. S4*A*). We found that the conditioned medium from TPMS scaffolds could increase the cell migration of HUVECs. Large Gaussian curvature could accelerate HUVEC migration (*SI Appendix*, Fig. S4 *A* and *C*). To further examine the effect of hMSCs’ paracrine on the formation of 3D tube-like structure of HUVECs, we performed the tube formation assay with hMSCs’ conditioned culture medium (*SI Appendix*, Fig. S4*B*). We found that the TPMS scaffolds supported more tubular structure formation, and the G6 groups presented 2.46-fold and 2.85-fold increase in total tube length and branching points compared to the G0 group after 6 h of culture (*SI Appendix*, Fig. S4 *D* and *E*). These results indicated that the hyperboloidal topology could enhance the angiogenic paracrine response of hMSCs and Gaussian curvature could regulate the paracrine secretion.

### Single-Cell Morphology on Hyperboloid Topology.

**Fig. 4.**
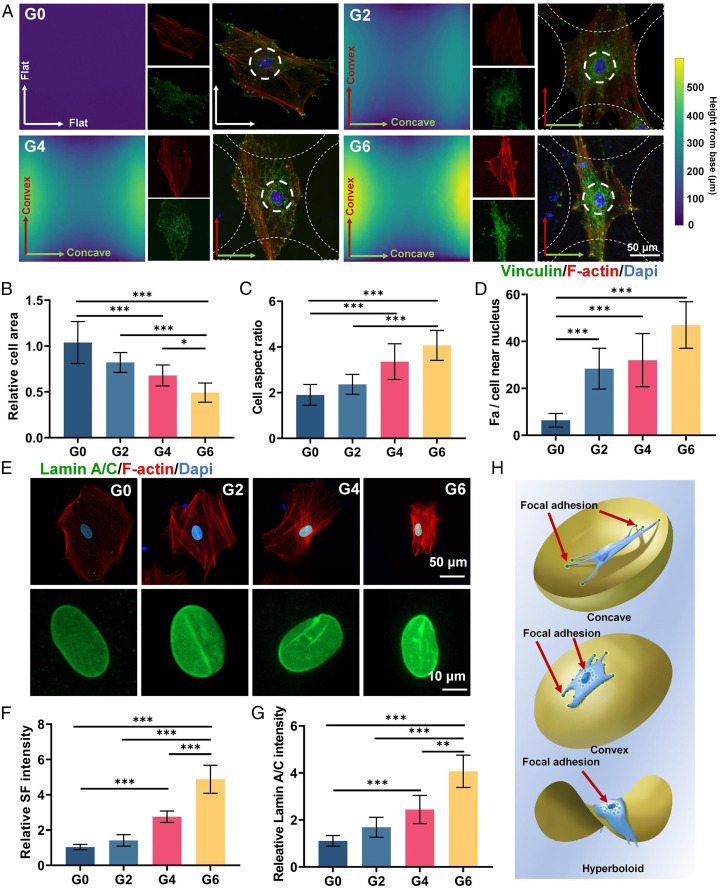
Effect of hyperboloid topology on SF, vinculin, and lamin A/C expression. (*A*) Immunofluorescence staining of vinculin (green), *F*-actin (red), and nuclei (blue) of hMSCs on hyperboloid surfaces. (*B*–*D*) Quantification of cell area, cell aspect ratio, and number of FAs near nuclei. (*E*) Immunofluorescence staining of lamin A/C (green), *F*-actin (red), and nuclei (blue) in hMSCs on hyperboloid surfaces. (*F* and *G*) Quantification of SF and lamin A/C intensity. (*H*) Schematic illustration of topology-induced cytoskeleton organization on concave, convex, and hyperboloid surfaces. The red arrows indicate the focal adhesion sites of the cells. Sample size *n* = 10 for all experiments by one-way ANOVA with Tukey’s post hoc test for multiple comparisons. Data are presented as mean ± SD. *P < 0.05, **P < 0.01, and ***P < 0.001 denote statistical significance.

The above results demonstrate that the TPMS scaffolds with hyperboloid topology could promote osteogenic differentiation and angiogenic paracrine response of hMSCs. To explore the possible underlying mechanism of this phenomenon, we printed four types of β-TCP hyperboloid surface scaffolds with corresponding Gaussian curvature of 3D TPMS scaffolds (0 [control], –2, –4, and –6 mm^−2^) to observe single-cell morphology on the hyperboloid topology. The printing direction was in alignment with the concave (*K*_2_ < 0) direction. We first applied SEM and 3D laser microscope scanning to analyze the hyperboloid topology of these scaffolds and found that all scaffolds were in accordance with our design (*SI Appendix*, Fig. S5*A*). We then seeded hMSCs on these hyperboloid surface scaffolds. We investigated the stress fibers (SFs) and the focal adhesion (FA) complex organization of hMSCs on different hyperboloid topology through *F*-actin and vinculin immunofluorescence staining (Fig. 4*A*). The G0 group presented randomized SF distribution with largest cell area and smallest cell aspect ratio, about 1.8 (Fig. 4 *B* and *C*). Interestingly, we observed obvious SF reorganization on the hyperboloid surfaces in the G2, G4, and G6 groups. The SF presented distinctly elongated orientation in the convex (*K*_1_ > 0) direction and presented contracted cell morphology in the concave (*K*_2_ < 0) direction. The G4 and G6 groups showed significantly decreased cell area and increased aspect ratio (3.36 and 4.06 for G4 and G6, respectively) (Fig. 4 *B* and *C*). Moreover, we found that the G0 group presented more vinculin (FA marker) in the periphery of the cells at the end of actin filament; however, the G2, G4, and G6 groups demonstrated more vinculin in the vicinity of the cell nucleus, indicating cellular attachment mode shift (Fig. 4 *A* and *D*). These results could be attributed to the cell contractility modulation on the hyperboloid surfaces. On the hyperboloid surfaces, the cells showed contracted cell shape in the concave (*K*_2_ < 0) direction, whereas they presented a snail-like configuration in the convex (*K*_1_ > 0) direction, with increased FA intensity near the nucleus. These results were consistent with previous finding that cells on concave (*K*_2_ < 0) surfaces underwent obvious cell contraction, while they tended to elongate radially on convex (*K*_1_ > 0) surfaces (10). We additionally found that the G2, G4, and G6 groups demonstrated significantly higher SF intensity compared to the G0 group, possibly due to the increased cell contractility to counteract the curvature-induced cytoskeleton reorganization (Fig. 4*F*). Such modulated cell contractility could further impose critical external forces to deform the cell nucleus through the interaction between the SF and the nucleus membrane to regulate the cell fate. We further performed the lamin A/C (nuclear membrane protein) staining to evaluate the nucleus mechanics. We found that the cell nuclei were flattened and smooth on the flat G0 surfaces but had shrunken bean-like morphology on the hyperboloid surfaces (G2, G4, and G6; Fig. 4*E*). Quantification of the lamin A/C signals also showed 4.06-fold higher intensity in the G6 group than in the G0 group, indicating the higher SF force imposed on the cell nuclei (Fig. 4*G*). These results collectively indicated that the hyperboloid topology could induce the cytoskeleton reorganization of hMSCs and further impose external forces to the cell nucleus and affect their morphology. Such nucleus morphology regulation could lead to a cascade of cell behavior modulation such as the aforementioned osteogenic differentiation and angiogenic paracrine of hMSCs (Fig. 4*H*).

To validate the robustness of the above results, we further performed a series of evaluations including the effect of nanopatterns resulting from layer-by-layer 3D printing and the block studies. We found our observed cell orientation along the convex direction was different from the previously reported tendency of hMSCs to orient along the concave direction on torus-shaped surfaces (37). This could be attributed to the different substrate materials with distinct stiffness (e.g., β-TCP and polydimethylsiloxane). Since the nanopatterns resulting from layer-by-layer 3D printing may affect the cell morphology and cell mechanics, we further printed the β-TCP hyperboloid surface scaffolds with a perpendicular printing orientation (aligned with the convex [*K*_1_ > 0] direction) to exclude possible effects (38). The SEM and 3D laser microscope scanning analysis confirmed the structural integrity of the scaffolds with different printing orientation (*SI Appendix*, Fig. S5*B*). Afterward, we seeded hMSCs on these hyperboloid surfaces and found the hMSCs also presented similar morphology with elongated orientation in the convex (*K*_1_ > 0) direction and contracted cell morphology in the concave (*K*_2_ < 0) direction (*SI Appendix*, Fig. S6*A*). This result indicated the cell morphology was irrelevant to the layer-by-layer nanopatterns. With the increase of Gaussian curvature, we also observed the increase in the cell aspect ratio and decrease in the cell area (*SI Appendix*, Fig. S6 *C* and *D*). In addition, we also observed the cell nucleus morphology to validate the effect of the nanopatterns on nucleus mechanics. We found the G6 groups also presented more vinculin expression in the vicinity of the cell nucleus and increased lamin A/C expression with shrunken nuclei deformation (*SI Appendix*, Fig. S6 *B*, *E*, and *G*). In all, these results showed the consistent cell morphology and cell mechanics on β-TCP hyperboloid surface scaffolds with two perpendicular printing orientations, which could be caused by the effect of the hyperboloid topology instead of the nanopatterns generated by the 3D printing process.

Moreover, to further validate whether such hyperboloid topology could induce the cytoskeleton reorganization of hMSCs, impose external forces to the cell nucleus, and affect their morphology, we examined the cell morphology with the treatment of inhibitors in terms of the FA organization, SF formation, and contraction. In the cell–ECM interaction, the cells could sense the ECM through the integrin engagement to mediate the FA organization and SF formation and contraction (39). In this regard, we first adopted the PF-573228 to inhibit the focal adhesion kinase (FAK), which plays a critical role in the integrin-mediated FA organization (40, 41). We found that the cells on the hyperboloid scaffolds treated with PF-573228 presented a significant decrease in cell aspect ratio in the convex (*K*_1_ > 0) direction and increased cell area (*SI Appendix*, Fig. S7 *A*, *C*, and *D*). The formation of the actin filament was drastically disrupted. The vinculin staining for FA evaluation further revealed that the accumulation effect of FA in the vicinity of the cell nucleus induced by the hyperboloid topology had vanished (*SI Appendix*, Fig. S7*E*). Moreover, we found that there was no obvious nucleus deformation in all groups with decreased lamin A/C expression after PF-573228 treatment (*SI Appendix*, Fig. S7 *B* and *G*). These results confirmed that the cytoskeleton reorganization on the hyperboloid topology was exhibited through integrin-mediated FA reorganization to regulate the cell morphology. In addition, we hypothesized that the downstream SF of the reorganized FA could impose external forces to the cell nucleus. We further used blebbistatin to inhibit the myosin II, which is the motor protein responsible for SF contractility and the force imposed on nucleus (42). With a low dose of blebbistatin for 3 d, we found that the cells on the hyperboloid scaffolds maintained an elongated morphology with actin filament formation despite the compromised cell aspect ratio and increase in cell area (*SI Appendix*, Fig. S8 *A*, *C*, and *D*). We could also observe the FA accumulation in the vicinity of the cell nucleus (*SI Appendix*, Fig. S8*E*). Interestingly, we found the nucleus morphology was kept unchanged with comparable lamin A/C expression among all groups, indicating the decrease in SF contractility substantially reduced the force applied to the nucleus (*SI Appendix*, Fig. S8 *B* and *G*). In all, we confirmed our speculation that the hyperboloid topology could induce FA reorganization as well as SF formation and contraction to modulate cytoskeleton, which can further impose external forces to the cell nucleus, leading to cell fate regulation.

### Bioinformatic Analysis of hMSCs on TPMS Scaffolds.

**Fig. 5.**
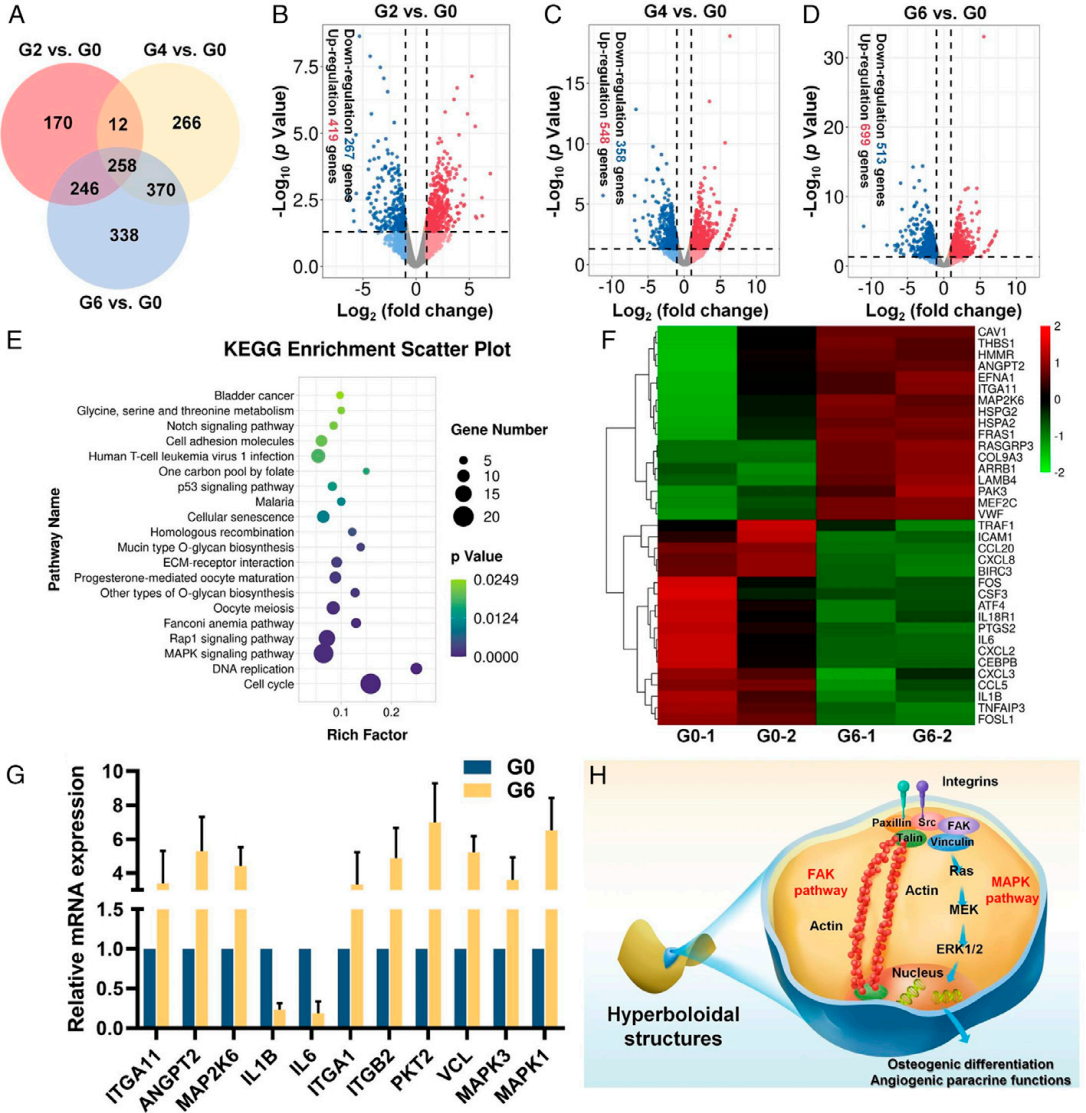
Bioinformatic analysis of gene expression of hMSCs on different TPMS scaffolds. (*A*) Venn diagram illustration of the DEGs between the TPMS scaffolds (G2, G4, and G6) and the conventional truss scaffold (G0). (*B–D*) Volcano plots of transcriptomic analysis of DEGs in (*B*) G2 versus G0, (*C*) G4 versus G0, and (*D*) G6 versus G0. (*E*) Up-regulated enriched Kyoto Encyclopedia of Genes and Genomes pathways of G6 versus G0. (*F*) Heatmap evaluation of DEGs involved in ECM–receptor interaction, cell adhesion molecule, MAPK, and proinflammatory signaling pathways. (*G*) Relative messenger RNA expression evaluation of targeted genes via qRT-PCR. (*H*) Schematic illustration of potential integrin-mediated FAK and MAPK pathway activation mechanism of hMSCs’ osteogenic differentiation and angiogenic paracrine response on the hyperboloidal structure.

To reveal the underlying mechanism of how hyperboloid surfaces affect the osteogenic differentiation and angiogenic paracrine response of hMSCs, we then performed the transcriptomic analysis of hMSCs cultured on various TPMS scaffolds with various Gaussian curvatures (G0, G2, G4, and G6). Pearson correlation analysis revealed that the correlation coefficients of all samples were within an acceptable range (>0.92), indicating satisfactory sample stability (*SI Appendix*, Fig. S9). Then, the differential gene expression between the two groups was evaluated by edgeR analysis. Compared with the G0 group, hMSCs cultured on G2, G4, and G6 groups presented a wide range of differential gene expressions (686 genes for G2 vs. G0, 906 genes for G4 vs. G0, and 1,212 genes for G6 vs. G0), with 258 intersecting genes (Fig. 5*A*). The volcano plots additionally showed 419 up-regulated and 267 down-regulated genes (G2 vs. G0), 548 up-regulated and 358 down-regulated genes (G4 vs. G0), and 699 up-regulated and 513 down-regulated genes (G6 vs. G0) (Fig. 5 *B*–*D*). The above results indicated the Gaussian curvature–dependent gene expression changes of hMSCs. Based on these results, we selected the G6 group with the most differentially expressed genes for further evaluation. We adopted the differentially expressed genes (DEGs) for Gene Ontology database evaluation, which included biological process, molecular function, and cellular component. The significantly enriched Gene Ontology terms (*P* < 0.05, G6 vs. G0) were collected, and we found some up-regulated terms including cell proliferation, cytoskeleton organization, and cell adhesion were correlated with the cytoskeleton reorganization (*SI Appendix*, Fig. S10 *A* and *B*). We then performed the Kyoto Encyclopedia of Genes and Genomes pathway analysis of the DEGs, which revealed the down-regulation of proinflammatory pathways including the tumor necrosis factor signaling pathway and interleukin 17 signaling pathway, as well as up-regulation of ECM–receptor interaction and mitogen-activated protein kinase (MAPK) signaling pathway related to topological modulation (Fig. 5*E* and *SI Appendix*, Fig. S11). In addition, the heatmap of DEGs in these pathways showed that a series of genes were significantly down-regulated (e.g., IL1B, IL6, CSF3) or up-regulated (e.g., ITGA, MAP2K6, ANGPT2) (Fig. 5*F*). To validate these gene expression data, we further performed qRT-PCR analysis and discovered that the down-regulated and up-regulated targeted gene expression are consistent with the RNA sequencing results (Fig. 5*G*). We found the expression of ITGA1, ITGB2, PKT2, VCL, MAPK3, and MAPK1 genes in G6 were significantly higher than that of the G0 group (Fig. 5*G*).

These results collectively indicated a potential integrin-mediated FAK and MAPK pathway activation mechanism for the coupled osteogenesis–angiogenesis. The integrin-mediated FAK pathway has been widely reported in the osteogenic differentiation of stem cells and therefore may provide an insight into osteogenesis promotion of the TPMS scaffolds (43). The transcriptomic analysis also revealed the possible MAPK pathway activation, which could be attributed to the increased integrin expression and modulated focal adhesion formation. The MAPK pathway is widely reported to direct stem cell differentiation toward osteoblasts (44). More importantly, activation of the MAPK pathway is responsible for the angiogenic paracrine effects of hMSCs on the angiogenesis of HUVECs (45, 46). Therefore, we hypothesized that hyperboloid topology could regulate the expression of integrin to modulate the FA complex organization (e.g., vinculin) and SF formation, to finally impose mechanical force on the nuclei to activate the FAK pathway, and to modulate cell fates (e.g., osteogenesis). Simultaneously, the FA complex activated downstream Ras and ERK 1/2 in the MAPK pathway (Fig. 5*H*). Finally, these activated pathways synergistically enhanced the osteogenic differentiation and angiogenic paracrine response of hMSCs to achieve osteogenesis–angiogenesis coupling. To validate such hypotheses, we also tested the effect of inhibitors of these pathways on the cell fates of the G6 group, which presented the most differentially expressed genes compared with the G0 group. We found FAK inhibitor PF-573228 in FAK pathway could significantly decrease ALP activity and relative osteogenic gene expression (e.g., OCN, COL-1) (*SI Appendix*, Fig. S12). In addition, the ERK 1/2 inhibitor FR180204 in the MAPK pathway could substantially decrease the angiogenic paracrine response of hMSCs, as demonstrated by the tube formation assay and relative angiogenic gene expression (e.g., VEGF-A, Ang-1) (*SI Appendix*, Fig. S13). Altogether, we speculated this FAK and MAPK pathway activation mechanism is responsible for the hMSCs’ osteogenic differentiation and angiogenic paracrine response on the hyperboloidal structure.

### In Vivo Osteogenesis Efficacy of TPMS Scaffolds.

**Fig. 6.**
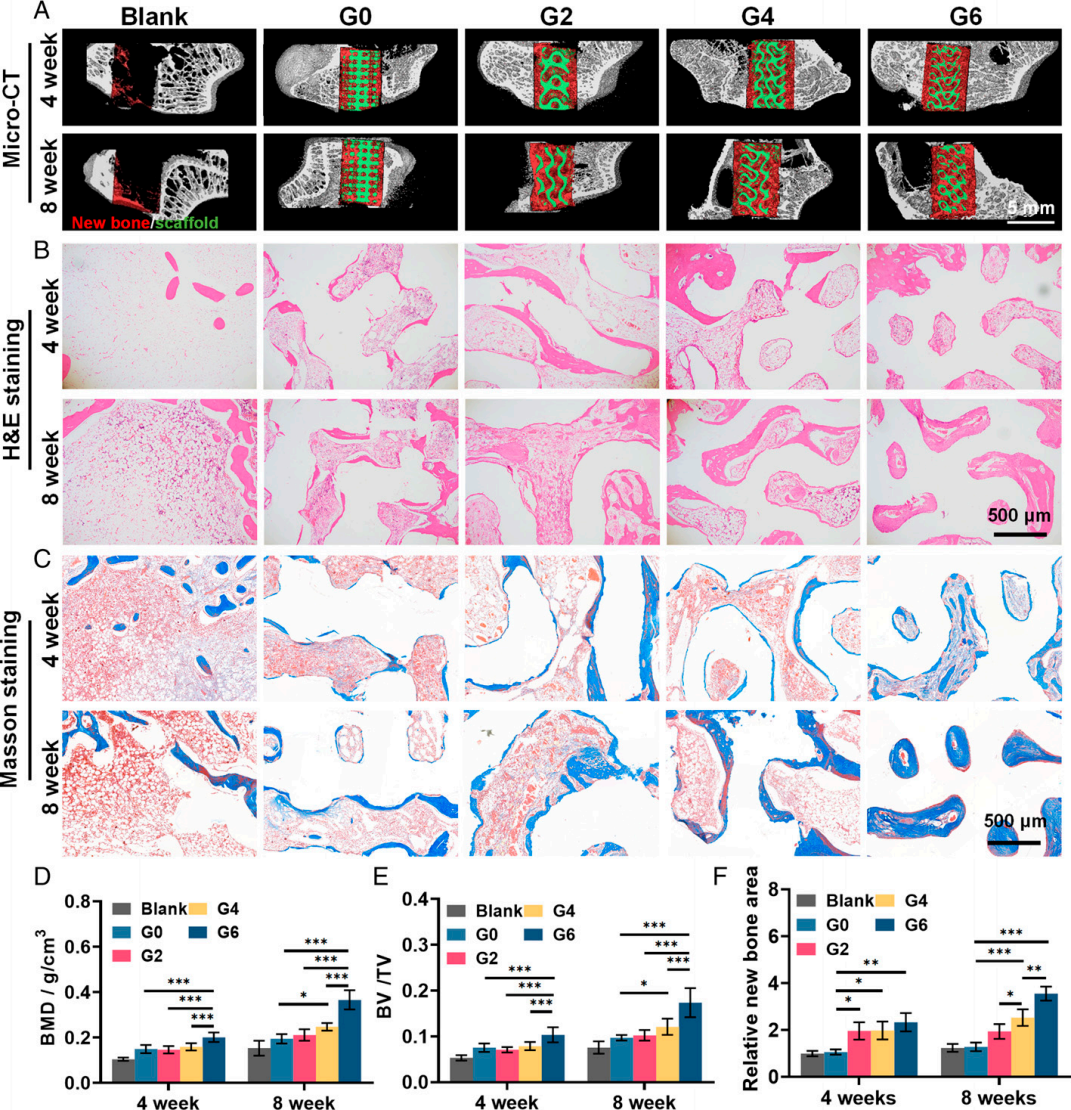
In vivo osteogenesis efficacy of 3D TPMS scaffolds in a rabbit femur defect model. (*A*) 3D reconstructed images of micro-CT scanning at 4 and 8 wk; red and green color denote the new bone tissue and scaffolds, respectively. (*B*) Hematoxylin and eosin staining and (*C*) Masson’s trichrome staining of the regenerated bone tissue. (*D* and *E*) Quantification of (*D*) BMD and (*E*) BV/TV via micro-CT evaluation. (*F*) Quantification of new bone area in Masson’s trichrome staining. Sample size *n* = 6 for all experiments by two-way ANOVA with Tukey’s post hoc test for multiple comparisons. Data are presented as mean ± SD. *P < 0.05, **P < 0.01, and ***P < 0.001 denote statistical significance.

To investigate the therapeutic efficacy of TPMS scaffolds on bone repair in vivo, we further established a rabbit femoral defect model (*SI Appendix*, Fig. S14*A*). Four kinds of scaffolds were adopted, including the TPMS scaffolds with Gaussian curvatures of –2, –4, or –6 mm^−2^ (denoted as G2, G4, and G6) and conventional truss scaffolds with 0 Gaussian curvature (denoted as G0) as control. After 4 and 8 wk of implantation, the samples were collected, and all scaffolds were found enveloped in soft tissue without obvious inflammation (*SI Appendix*, Fig. S14*B*). The micro-CT reconstruction was used to evaluate the new bone formation (red) within scaffolds (green) (Fig. 6*A*).

The TPMS groups presented more new bone formation than the control G0 group. Statistical analysis further revealed that the G6 group presented significantly higher bone mineral density (BMD; 0.201 ± 0.021 g/cm^3^ and 0.365 ± 0.042 g/cm^3^) and bone volume/total volume value (BV/TV; 0.103 ± 0.017 and 0.174 ± 0.032) than other groups after both 4 and 8 wk (Fig. 6 *D* and *E*). In histological results of hematoxylin and eosin staining, tissue ingrowth significantly increased in the TPMS groups compared to the control group, demonstrating that the TPMS scaffolds with the continuous curved surface could facilitate neotissue infiltration (Fig. 6*B*). The amount of the newly formed bone increased with the Gaussian curvatures, and the G6 group presented the highest amount of new bone tissue. Meanwhile, in Masson’s trichrome staining, we found that the new bone tissue in the G6 group was higher in density than other groups, and the new bone tissue in the G6 group filled all the space in the scaffolds after 8 wk (3.12 folds compared to G0), emphasizing its optimal potential in supporting the new bone formation (Fig. 6 *C* and *F*).

We designed our TPMS scaffolds with different Gaussian curvatures by scaling the unit cell size with consistent porosity. However, as it is impossible to create scaffolds of varied Gaussian curvature with consistent porosity, surface area, and pore size, we acknowledge that there may be additional factors affecting the in vivo efficacy of the TPMS scaffolds. It has been reported that pore size relates to bone regeneration via effects on cell infiltration and adhesion. Small pores could lead to compromised cell infiltration, while overly large pores could decrease the cell adherence. The optimal range of pore size lies roughly within 200–1,200 µm (47, 48). In addition, bone remodeling is typically a layer-by-layer process on biomaterial surfaces, indicating that bone tissue deposition could benefit from the larger surface area (49). The increased surface area has also demonstrated higher bone regeneration in several studies (50–52). To this end, we consciously designed the G0 control group with comparable surface area with the G4 group and pore size with the G6 group to give more insight into the in vivo efficacy of bone regeneration of the TPMS scaffolds. In the in vivo evaluation, we observed that the G4 group expressed significantly more bone regeneration than G0, despite both exhibiting similar porosity and surface area. Moreover, the G6 group presented a significantly higher in vivo efficacy of bone regeneration than the G0 group with the same porosity and pore size. In addition, with the increase in Gaussian curvature, the efficacy of bone regeneration was substantially boosted with significantly increased BMD, BV/TV, and new bone formation. Moreover, to give more insight into the effect of Gaussian curvature on bone regeneration, we further examined the spatial correlation between local Gaussian curvature on each TPMS surface and the new bone formation since each TPMS scaffold contains broad variation in Gaussian curvatures. After overlapping the Gaussian curvature distribution and the new bone formation of each scaffold at 4 and 8 wk, we found that there was more pronounced new bone formation (red) in the regions with high Gaussian curvature (blue) than in regions with low Gaussian curvature (yellow) in each scaffold (*SI Appendix*, Fig. S15*A*). Quantification of the new bone formation against the local Gaussian curvature for G2, G4, and G6 scaffolds further supports the obvious correlation between Gaussian curvature and bone regeneration (*SI Appendix*, Fig. S15 *B*–*D*). These results collectively indicate that surface area and pore size were not the principal factors modulating bone regeneration in our study, while evidence showed that Gaussian curvature was the driving factor modulating bone regeneration. More importantly, in single-cell morphology observation, we have demonstrated such hyperboloid topology could modulate cell morphology and nuclei biomechanics to regulate cell fate. The in vitro osteogenesis studies also revealed that TPMS scaffolds presented higher osteogenic potential than the control G0 group. Notably, these in vitro evaluations are not likely to be affected by pore size and surface area as the cell density was nearly the same for all TPMS scaffolds and the osteogenesis evaluations were normalized by cell number. Therefore, we speculate that our claim that Gaussian curvatures altered enhanced bone regeneration stands for the in vivo evaluation.

### In Vivo Angiogenesis Efficacy of TPMS Scaffolds.

To further evaluate the effect of TPMS scaffolds on angiogenesis, we subcutaneously implanted TPMS scaffolds in male C57 mice. This model has been widely used for neovascularization evaluation of scaffolds (53). As expected, the TPMS groups showed more neotissue ingrowth, with better integration with the surrounding host tissue after 35 d. The G6 group presented most infiltrated fibrous tissues and inside neovasculature throughout the scaffolds, indicating its potential to support angiogenesis and neovascular infiltration (*SI Appendix*, Fig. S9*A*). To computationally analyze the number and morphology of blood vessels, we further applied CD31 fluorescence staining (vascular formation marker) and found that the CD31 intensity of the G4 and G6 groups was higher than that of other groups (2.4- and 3.25-fold higher, respectively, than that of G0) (*SI Appendix*, Fig. S9 *B* and *C*). These results indicated that the TPMS scaffolds could promote neovascular formation and infiltration which are critical for bone regeneration.

## Conclusion

In this study, we designed TPMS scaffolds with various Gaussian curvatures (–2, –4, and –6 mm^−2^) and successfully fabricated the scaffolds comprised of body-inherent β-TCP by using stereolithography-based 3D printing and sintering. We performed a thorough optimization of printing and sintering parameters, achieving control over the 3D scaffold structures with excellent accuracy and reproducibility. With the same porosity (∼60%) of interconnected pores, we found that the TPMS scaffolds boasted significantly higher compressive strength (∼60 MPa) compared to the control conventional truss scaffold (∼40 MPa). The impressive mechanical property is attributed to the smoothly curved surfaces, which minimize stress concentration. Additionally, the TPMS scaffolds can well support the survival and proliferation of hMSCs and substantially enhance the osteogenic differentiation and angiogenic paracrine function of hMSCs. The hMSCs on the hyperboloid surfaces showed a contracted cell shape in the concave (*K*_2_ < 0) direction, whereas they presented a snail-like configuration in the convex (*K*_1_ > 0) direction because of the curvature-induced stress fiber reorganization. Transcriptomic analysis revealed integrin-mediated FAK and mitogen activated protein kinase (MAPK) pathway activation of the hMSCs on the TPMS scaffolds. We theorize that the wavy TPMS structure deforms the cell nuclei to a shrunken bean-like morphology through integrin-mediated stress fiber reorganization, directing cell fate toward osteogenesis and angiogenesis. Lastly, the rabbit femoral defect model demonstrated the distinct performance of our TPMS scaffolds in terms of new bone formation. The mouse subcutaneous implantation model further validated the scaffolds’ substantial potential in supporting tissue infiltration and neovascularization.

This study shepherded osteogenic differentiation and angiogenic paracrine of hMSCs through biomimicking hyperboloidal topography–induced nuclear deformation and cytoskeleton reorganization for expedited bone regeneration. Our TPMS bone scaffolds have high porosity and interconnectivity, improved mechanical strength, and excellent biocompatibility. They can well support osteogenesis in vitro and in potential as a head start toward a safer and more efficient bone graft with notable clinical translation potential. Our design will also provide guidelines to design simple, efficient, and personalized bone grafts with simultaneous osteogenesis and angiogenesis. The TPMS concept is also transferable toward designing other bone implants, such as metal or polymer prostheses.

## Materials and Methods

Details of the design, fabrication, and characterization of the TPMS scaffolds are described in *SI Appendix*. The in vitro and in vivo experimental details including cytocompatibility evaluation, effect of TPMS scaffolds on osteogenic differentiation and angiogenic paracrine of hMSCs, and animal experiments are also described in *SI Appendix*. All animal evaluation was performed with approval from the Ethics Committee of the Hong Kong Polytechnic University (21-22/40-BME-R-CRF).

## Supplementary Material

Supplementary File

## Data Availability

All study data are included in the article and/or *SI Appendix*. Raw data for transcriptomic analysis were deposited under NCBI BioProject PRJNA763989 (54).
