# Supplementary material for: Gaussian curvature–driven direction of cell fate toward osteogenesis with triply periodic minimal surface scaffolds
Source: Proc Natl Acad Sci U S A. 2022 Oct 3;119(41):e2206684119. doi: 10.1073/pnas.2206684119 (PMC9564829; doi:10.1073/pnas.2206684119)
Supplement: Supplementary File [file pnas.2206684119.sapp.pdf]

PNAS

Supplementary Information for

Gaussian curvature-driven direction of cell fate towards osteogenesis with triply periodic minimal surface scaffolds

Yuhe Yang[a], Tianpeng Xu[a], Ho Pan Bei[a], Lei Zhang[b], Chak-Yin Tang[c], Ming Zhang[a], Chenjie Xu[d], Liming Bian[e,f], Kelvin Wai-Kwok Yeung[g]*, Jerry Ying Hsi Fuh[h]*, Xin Zhao[a]*

[a]Department of Biomedical Engineering, The Hong Kong Polytechnic University; Hung Hom, Hong Kong SAR, China

[b]Department of Mechanical and Aerospace Engineering, Hong Kong University of Science and Technology; Kowloon, Hong Kong SAR, China

[c]Department of Industrial and Systems Engineering, The Hong Kong Polytechnic University; Hung Hom, Kowloon, Hong Kong SAR, China

[d]Department of Biomedical Engineering, City University of Hong Kong; Kowloon, Hong Kong SAR, China

[e]School of Biomedical Sciences and Engineering, South China University of Technology; Guangzhou, 510006, China

[f]National Engineering Research Center for Tissue Restoration and Reconstruction, South China University of Technology, Guangzhou, 510006, China

[g]Department of Orthopaedics & Traumatology, Li Ka Shing Faculty of Medicine, The University of Hong Kong; Pokfulam, Hong Kong SAR, China

[h]Department of Mechanical Engineering, National University of Singapore; 117575, Singapore

[1]Y.Y. and T.X. contributed equally to this work

*To whom correspondence may be addressed

Email: wkkyeung@hku.hk (K.W.K Y.); jerry.fuh@nus.edu.sg (Y.H. F.); xin.zhao@polyu.edu.hk (X.Z.)

This PDF file includes:

Supplementary text: Materials and Methods
Figure S1 to S16
Tables S1 to S3
SI References

Materials and Methods

Design, fabrication, and characterization of TPMS scaffolds: TPMS exhibit zero mean curvature and non-positive Gaussian curvature at all points. The morphometric analysis of trabecular bone indicated its hyperbolic geometries (negative Gaussian curvature) which is consistent with the geometry of TPMS.(1) Gyroid-type TPMS was adopted in this study, and the sheet Gyroid scaffolds were modelled using the periodic nodal approximation of TPMS with the region of $\Phi \leq 0$ representing the solid domain of the scaffolds:

$$\Phi = (\cos(\omega x)\sin(\omega y) + \cos(\omega y)\sin(\omega z) + \cos(\omega z)\sin(\omega x))^2 - c^2$$

where $\omega = 2\pi/L$, and L is the unit cell size. The parameter c controls the porosity of the scaffolds. In this study, the porosity was fixed at 60%, corresponding to c = 0.6164. Previously, it has been reported that the curvature of a 3D surface scale is linear with $1/L^2$, as indicated by the dimensional analysis, implying a simple way to tune the curvature of scaffolds is by changing the unit cell size.(2) To match the Gaussian curvature (K) of natural trabecular bones, sheet Gyroid scaffolds with the average Gaussian curvature ranging from -2, -4, -6 mm^{-2} were designed by varying the cell size (denoted as G2, G4, and G6 respectively). The average principal radii (r) of the scaffolds could be calculated based on the formula $r = \pm\sqrt{-1/K}$. Thus, the three TPMS scaffolds could be described as G2 (K = -2 mm^{-2}, r = ± 0.707 mm), G4 (K = -4 mm^{-2}, r = ± 0.5 mm), G6 (K = -6 mm^{-2}, r = ± 0. 408 mm). For each average Gaussian curvature, the specimens were designed as 7 × 7 × 2 mm^3 cuboids for *in vitro* experiments and Ø 5mm × 8mm cylinders for *in vivo* experiments. All geometric models of sheet Gyroid scaffolds were generated by in-house developed MATLAB scripts, with stereolithography (STL) files as output for 3D printing. Scaffolds with hyperboloid surfaces with the same Gaussian curvature (-2, -4, and -6 mm^{-2}) at the saddle point were also designed and fabricated to examined the effect of Gaussian curvature on single cell behaviors including cell morphology re-organization, vinculin and Lamin A/C expression.

A commercial β-TCP slurry from Lithoz GmbH containing β-TCP nanoparticles (50 wt.%), 1,6-hexanediol diacrylate based resin (49 wt.%) and photoinitiator (camphorquinone, 1 wt.%) were used to fabricate the designed TPMS scaffolds using the stereolithography-based printer (Lithoz CeraFab 8500, Austria). The layer thickness was set as 25 µm and the exposure time was 15 sec for each layer with 200 mW/cm^2 intensity. After the printing process, the cured scaffolds were clean with acetone (Sigma-Aldrich, China), followed by the debinding (205°C with 1°C/min heating rate and 16 h dwell time) and sintering process (1200°C with 1°C/min heating rate and 4 h dwell time) in ambient air. The micro-CT (Bruker, USA) and SEM (Tescan VEGA3, Czech) were used to characterize the structure and morphology of the printed scaffolds.(3) In addition, a 3D laser microscope (Keyence VK-X200, Japan) was applied to analyze the morphology and curvature of the printed hyperboloidal surface scaffolds (n = 3). Compressive test (Instron 5982, USA) was performed to analyze the mechanical properties of the 3D TPMS scaffolds (Ø5mm × 8mm) with 1 mm/min loading rate until specimen failure according to ASTM C1424 standard.(4, 5) Linear elastic finite element analysis was conducted to study the stress distributions of scaffolds with Abaqus 2019. The unit cells of the sheet Gyroid and truss scaffolds were meshed using linear tetrahedral (C3D4) and linear hexahedral (C3D8R) elements, respectively. The β-TCP ceramic was modelled as a linear elastic material with the Young's modulus of 145 GPa and Poisson's ratio of 0.22.(6, 7) The unit cell models were loaded by a small uniaxial loading under periodic boundary conditions. The von Mises stresses were extracted to evaluate stress concentration of all scaffolds.

Cytocompatibility evaluation: hMSCs (Cyagen, China, passage 3-5) was used to examine the cytocompatibility of the TPMS scaffolds. The cell culture medium was prepared by α-MEM (Gibco, China) supplemented with 10% fetal bovine serum (Gibco, China) and 1% penicillin/streptomycin (Gibco, China). The scaffolds were disinfected with 70% ethyl alcohol solution and washed with phosphate-buffered saline (PBS, Sigma-Aldrich, China). The cytocompatibility of the scaffolds was evaluated by seeding hMSCs onto the scaffolds. After putting different scaffolds (7 × 7 × 2 mm^3) in the bottom of a 24 well-plate, 1 mL hMSC suspension (2×10^4 cells/mL) was added onto the scaffolds and pipetted for three times to ensure the cell suspension can permeate into the scaffolds. After 6 h of cell seeding, the well-plates were changed to remove the unattached cells on the TPMS

scaffolds. After 1, 3 and 7 days of incubation, the cell viability was assessed by Live/Dead kit (Thermo Fisher, China) according to the manufacturer's protocol. The quantification of cell viability was determined by the ratio of the viable cells to all cells in eight randomly selected images. In addition, the cell number on scaffolds at different time points was evaluated by dissociating the cells with Trypsin-EDTA solution (Thermo Fisher, China) and counted by the hemocytometer (Thermo Fisher, China). The cell density was calculated through normalizing the cell number by the surface area of the scaffolds.

Effect of TPMS scaffolds on osteogenic differentiation of hMSCs: The osteogenic differentiation of hMSCs on TPMS scaffolds was evaluated by seeding hMSCs onto the scaffolds by pipetting 1 mL hMSC suspension (4×10^4 cells/mL) on the scaffolds in a 24-well plate. The cells were cultured in osteogenic induced medium (Cyagen, China) containing 0.25 mM ascorbate, 10 mM β-glycerol phosphate, 0.1 μM dexamethasone and 10% FBS in α-MEM. ALP activity of the hMSCs seeded onto different scaffolds was firstly studied. Briefly, after 7 and 14 days of incubation, the cells were fixed by paraformaldehyde (Bioshark, China) for 10 min followed by staining with the BCIP/NBT working solution (Sigma-Aldrich, China). The ALP activity of the hMSCs was quantified by the ALP activity kit (Beyotime, China) with total protein normalization. The ARS staining (Solarbio, China) was used to study the mineralization of the hMSCs. After cell fixation, the ARS staining solution was added to the samples for 20 min. Then, the samples were gently washed by deionized water until the color vanished. All images were taken using an optical microscope (Nikon, Japan). Afterwards, the scaffolds were destained with 10% cetylpyridinium chloride (Sigma-Aldrich, China) for 1 h. The extracts were collected and the absorbance at 562 nm was measured by a microplate reader (BioTek, US).(8) We also evaluated the OCN expression of hMSCs cultured on TPMS scaffolds. After the cell fixation and permeabilization, the primary antibodies of OCN (Abcam, China) were diluted with PBS (1:200) and incubated with cells overnight at 4°C. Then, the Alexa Fluor-coupled secondary antibody (Abcam, China) was diluted with PBS (1:200) and incubated with cells for 30 min at room temperature. The laser scanning confocal microscope (LCSM, Zeiss, German) was used for OCN immunofluorescence evaluation. We performed the qRT-PCR to analyze the osteogenic gene expression (ALP, OCN, Col-1 and RUNX2).(9) Briefly, total RNA was isolated from cells cultured on TPMS scaffolds using TRIzol reagent (Genstar, China). After generating the cDNA by reverse transcription, we performed the qRT-PCR by CFX 96 detection system (Bio-rad, USA). Primers of target genes were listed in Table S2.

Effect of TPMS scaffolds on angiogenic paracrine effect of hMSCs: To evaluate the angiogenic paracrine effect of hMSCs, the hMSC conditioned medium was firstly prepared.(10) Briefly, we pipetted 1 mL of hMSC suspension (4×10^4 cells/mL) onto TPMS scaffolds in a 24-well plate with the same protocol in cytocompatibility evaluation. The cells were cultured in a medium composed of 50% α-MEM and 50% Endothelial Cell Medium (ScienCell, China). After 72 h incubation, we collected the culture medium as the hMSC conditioned medium for further evaluation of angiogenesis of HUVECs. We firstly performed the wound healing assay according to a previous study.(11) Briefly, the HUVECs were seeded in six-well plates at a density of 5×10^4 cells/cm^2. After 24 h incubation, we scratched the cell monolayer with a p200 pipette tip and cultured the cells with the conditioned medium. The cells were stained with crystal violet for the wound healing evaluation after 24 h of incubation. The migration ratio (A) was obtained by the following equation: $A = (A_0 - A_1)/A_0 \times 100\%$, where A_0 and A_1 denoted the area of initial scratch and final scratch respectively. We then performed the tube formation assay according to our previous study.(12) Briefly, we seeded the HUVECs onto a Matrigel coated 24-well plate at a density of 5×10^4 cells/cm^2 with the hMSC conditioned medium. After 2 h and 6 h, we stained the HUVECs by Calcein AM (Thermo Fisher, China) and took photos by an inverted microscope (Nikon, Japan). We then analyzed the total length and average number of branching points by the Image J software (NIH, USA).

Underlying mechanism elucidation: To give insight of the possible mechanism of osteogenic differentiation and the angiogenic paracrine of hMSCs on TPMS scaffolds, we investigated the cell morphology and analyzed the cytoskeleton change of hMSCs cultured on hyperbolic surfaces with

the same Gaussian curvature of TPMS scaffolds. We firstly performed vinculin, *F*-actin and Lamin-A/C fluorescent staining according to a previous study.(12) In brief, after 3 days of incubation, cells seeded on the hyperboloid surfaces were fixed with Block buffer (Bioshark, China) and permeabilized by 0.1% Triton X (Beyotime, China) for 15 min. Afterwards, Lamin-A/C antibody (Abcam, UK), and vinculin antibody (Abcam, UK) were diluted with PBS (1:200) and then incubated with cells at 4°C overnight. *F*-actin was stained with Phalloidin 633 (Thermo Fisher, China) and cell nuclei were counterstained with DAPI (Thermo Fisher, China). All images were taken by LCSM and analyzed by image J software. To further demonstrate the effect of hyperboloid surface on cell morphology modulation, we treated the hMSCs cultured on hyperbolic surfaces with 10 μM PF-573228 (FAK inhibitor, MCE, USA) or 5 μM blebbistatin (myosin II inhibitor, MCE, USA) for 3 days, respectively. Then, we performed the immunofluorescence staining of vinculin, *F*-actin and Lamin-A/C to observe the cell morphology.

To evaluate the possible downstream signal pathways activated by the TPMS scaffolds, we additionally seeded hMSCs on TPMS scaffolds and performed the RNA sequence analysis. The total RNA from hMSCs was extracted using Trizol reagent (Beyotime, China) after culturing on TPMS scaffolds for 3 days. RNA-Seq libraries were prepared by NEBNextRUltraTM RNA Library Prep Kit.(9) The pooled library was sequenced by Illumina HiseqX-ten platform. The Gene Ontology (GO) enrichment and Kyoto Encyclopedia of Genes and Genomes (KEGG) analysis were performed using cluster Profiler R package. We then conducted the block experiments to further validate the effect of FAK and MAPK signal pathways on the osteogenic differentiation and the angiogenic paracrine action of hMSCs. Briefly, the hMSCs cultured on the G6 scaffolds were treated with 10 μM FAK inhibitor PF-573228 or 3 μM ERK 1/2 inhibitor FR180204 (MCE, USA). Then, the ALP staining and activity evaluation and relative expression of osteogenic genes (e.g., OCN, COL-1) were performed to characterize the effect of FAK inhibition on the osteogenesis of hMSCs on TPMS scaffolds. The FR180204 treated hMSCs were also used to study the effect of ERK 1/2 inhibition on the angiogenic paracrine response of hMSCs on TPMS scaffolds in terms of the tube formation and relative angiogenic gene expression (e.g., VEGF-A, Ang-1). The primers of genes for qRT-PCR verification were listed in Table S3.

Therapeutic efficacy of TPMS scaffolds on bone regeneration: All animal evaluation was performed with approval from the Ethics Committee of the Hong Kong Polytechnic University (21-22/40-BME-R-CRF). We firstly performed the rabbit femoral defect model to assess the therapeutic efficacy of bone regeneration of the TPMS scaffolds.(13) 60 New Zealand white rabbits (male, 2.5-3 kg) were adopted and randomly divided into five groups (blank, G0, G2, G4 and G6, *n*=6 per group for each time point). This is because the acceptable range of degree of freedom (DF) was between 10 and 20 and *n*=6 could satisfy the power analysis.(14) We then anesthetized the rabbits with pentobarbital sodium and created a 6-cm skin incision to expose the femoral condyle. Next, a dental drill was used to create a 5-mm diameter round defect on the exposed femoral condyle. We then implanted the TPMS scaffolds (Ø5 mm × 8 mm) and sutured the defect sites of the rabbits. After 4 and 8-week post-implantation, the rabbits were sacrificed (*n*=6 per group) with CO_2 suffocation and the femur samples were harvested. The high-resolution micro-CT (SkyScan 1176, Belgium) was used to analyze the bone regeneration using the scaffolds.(15) The scanning parameters were set as source voltage 80 kV, source current 124 μA, 0.5 mm Al filter and rotation step of 0.6°. The different thresholding ranges (threshold for new bone = 80 ~140, scaffold = 140) were applied to distinguish the new bone and the scaffold.(16, 17) 3D models were reconstructed by the NRecon software (Bruker, Belgium). Quantification results of the BMD and BV/TV were calculated by CTAn software (Bruker, Belgium). The reconstructed models of the new bones were further aligned with the as-designed TPMS scaffolds using the open-source mesh processing software Meshlab. To correlate the spatial distribution of the new bone formation and the local curvature of the scaffolds, the Gaussian curvature of the TPMS scaffolds was calculated based on the implicit representation of the TPMS scaffolds. For visualization, a slice of the scaffold rendered by its Gaussian curvature was plotted and the aligned new bone was overlapped on the scaffold using Matlab scripting. To further quantify the correlation between the new bone formation and the local curvature of the scaffolds, we calculated the volume of the new bone adjacent to the scaffold at all locations. For each point on the as-designed TPMS scaffolds, the Gaussian curvature at each point of the scaffold was calculated and linked with the adjacent new bone volume which was calculated by a cube with

a prescribed side length (200 μm). For each scaffold, we selected the inner regions of TPMS scaffolds as regions of interest (ROI) and grouped the data into 5 groups according to the curvature. Finally, we plotted the new bone volume normalized by the average volume of the zero-curvature group against the local Gaussian curvature. After the micro-CT analysis, the histology analysis was further performed. H&E staining and Masson's trichrome staining were performed to observe the formation of new bone tissues in the defect sites. The sample sectioning, staining and imaging was performed by Servicebio (China).

Meanwhile, we applied the mouse subcutaneous implantation model to validate the effect of TPMS scaffolds on neovascularization.(18) Briefly, 30 male BALB/c mice were randomly divided into five groups (blank, G0, G2, G4 and G6) and anesthetized by inhalational isoflurane. We then created a subcutaneous incision (6 mm) on the back of the mice with two Ø 5mm × 8 mm cylindrical scaffolds inserted under the skin and pushed to both sides of the incision. The mice were sacrificed at 35 days post-implantation. After removing the surrounding tissues, we prepared the samples for paraffin section and performed the H&E staining and CD31 immunofluorescence staining for evaluation of neovascularization. We observed the sections using fluorescence microscopy and assessed the fluorescence intensity by Image J software.

Statistical analysis: All tests were performed in quadruplicate unless otherwise indicated and the values were presented as mean ± standard deviation. One-way or two-way ANOVA by Tukey post-hoc was adopted to conduct statistical evaluation between each group with GraphPad Prism Software (GraphPad Software Inc.). A difference at $^{*}p < 0.05$, $^{**}p < 0.01$, or $^{***}p < 0.001$ was considered statistically significant.

Supplementary Figures

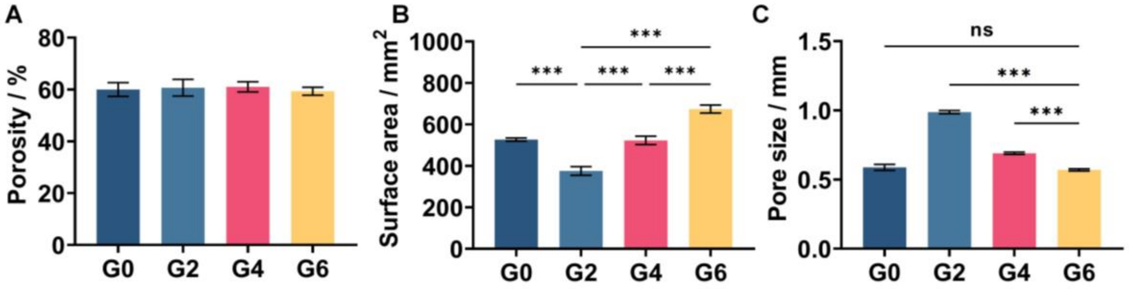

Fig. S1. Quantification of the (A) porosity, (B) surface area and (C) pore size of the 3D printed scaffolds. Data are presented as mean ± SD. ****p* < 0.001 denote the statistical significance. Please note that the G0 groups exhibited comparable surface area with the G4 group and pore size with the G6 group.

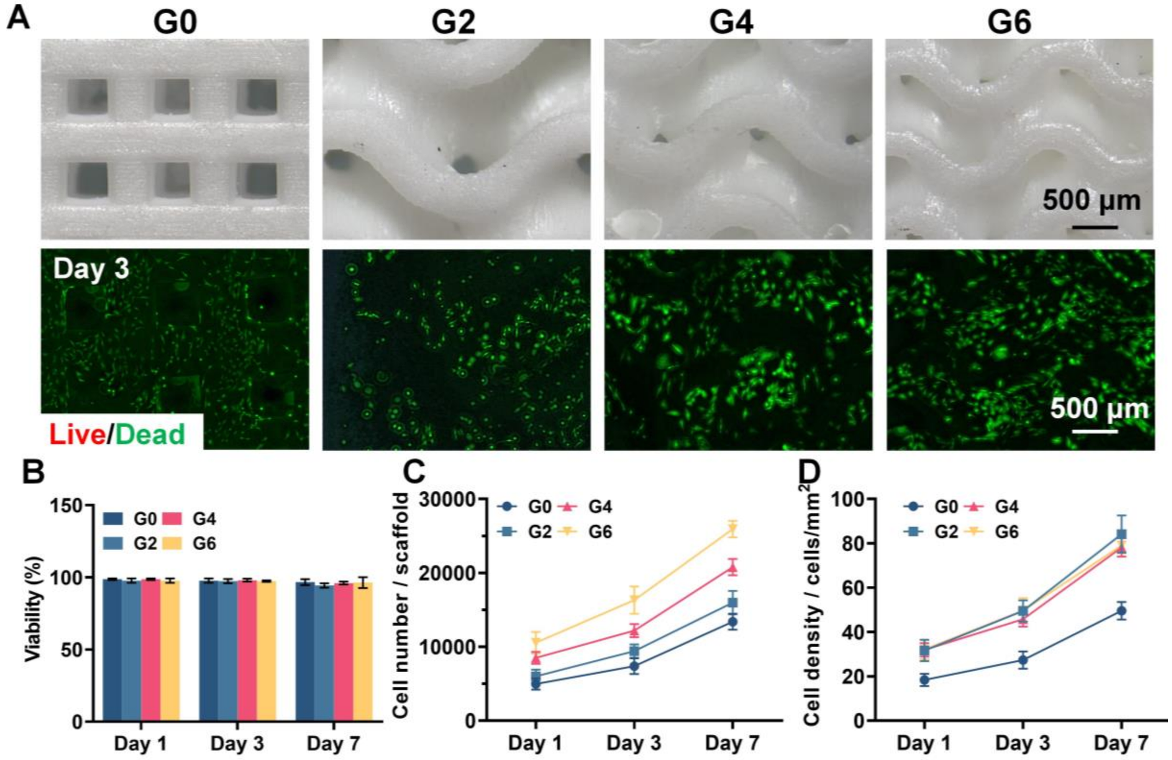

Fig. S2. Cytocompatibility of TPMS scaffolds. (A) Live/dead staining and (B) quantification of cell viability, (C) cell number / scaffold and (D) cell number / surface area of the TPMS scaffolds. Sample size *n* = 3 for all experiments. Data are presented as mean ± SD.

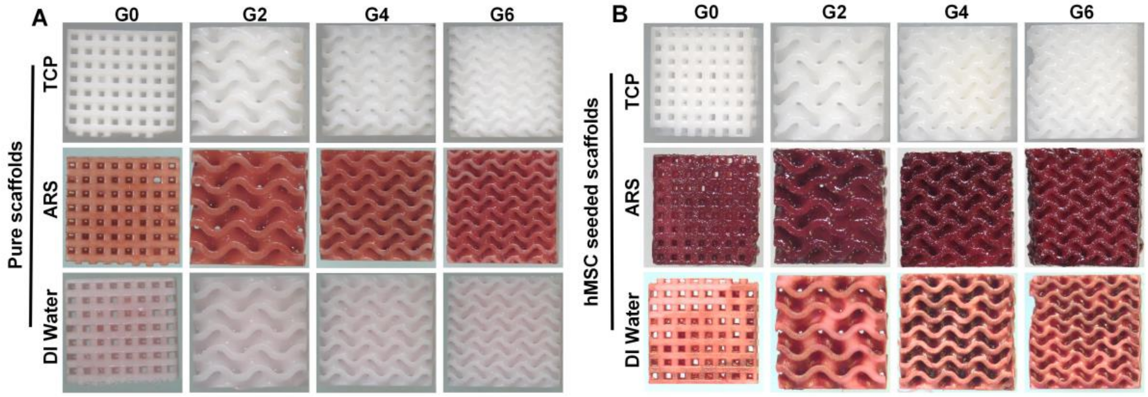

Fig. S3 Validation of ARS staining for evaluation of calcium nodule formation on the tricalcium phosphate based TPMS scaffolds. (A) ARS staining of the pure scaffolds without any cells. The red calcium nodule could be washed away with deionized (DI) water. (B) ARS staining of the pure scaffolds seeded with cells. The red calcium nodule retained after DI water wash.

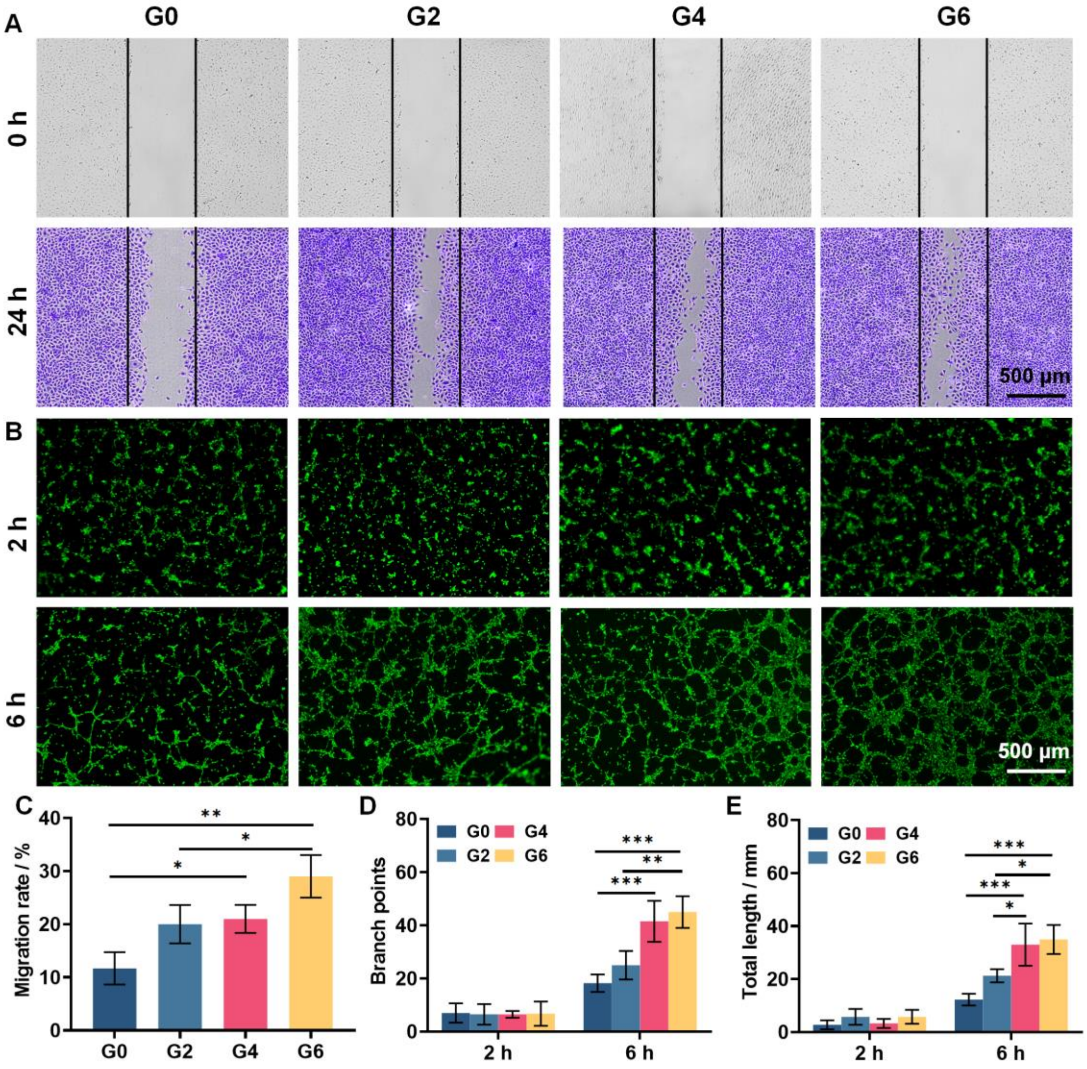

Fig. S4. Angiogenic paracrine function of hMSCs seeded on different TPMS scaffolds. (A) Wound healing evaluation of HUVECs; (B) Representative fluorescence images of HUVECs in tube formation assay after 2 hours and 6 hours of culture. (C) Migration rate of the HUVECs in wound healing assay; (C) Branching points and (D) total length of tube-like structure in tube formation assay. Sample size $n = 3$ for all experiments by a one-way or two-way ANOVA with a Tukey's post hoc test for multiple comparison. Data are presented as mean ± SD. *$p < 0.05$, **$p < 0.01$, and ***$p < 0.001$ denote the statistical significance.

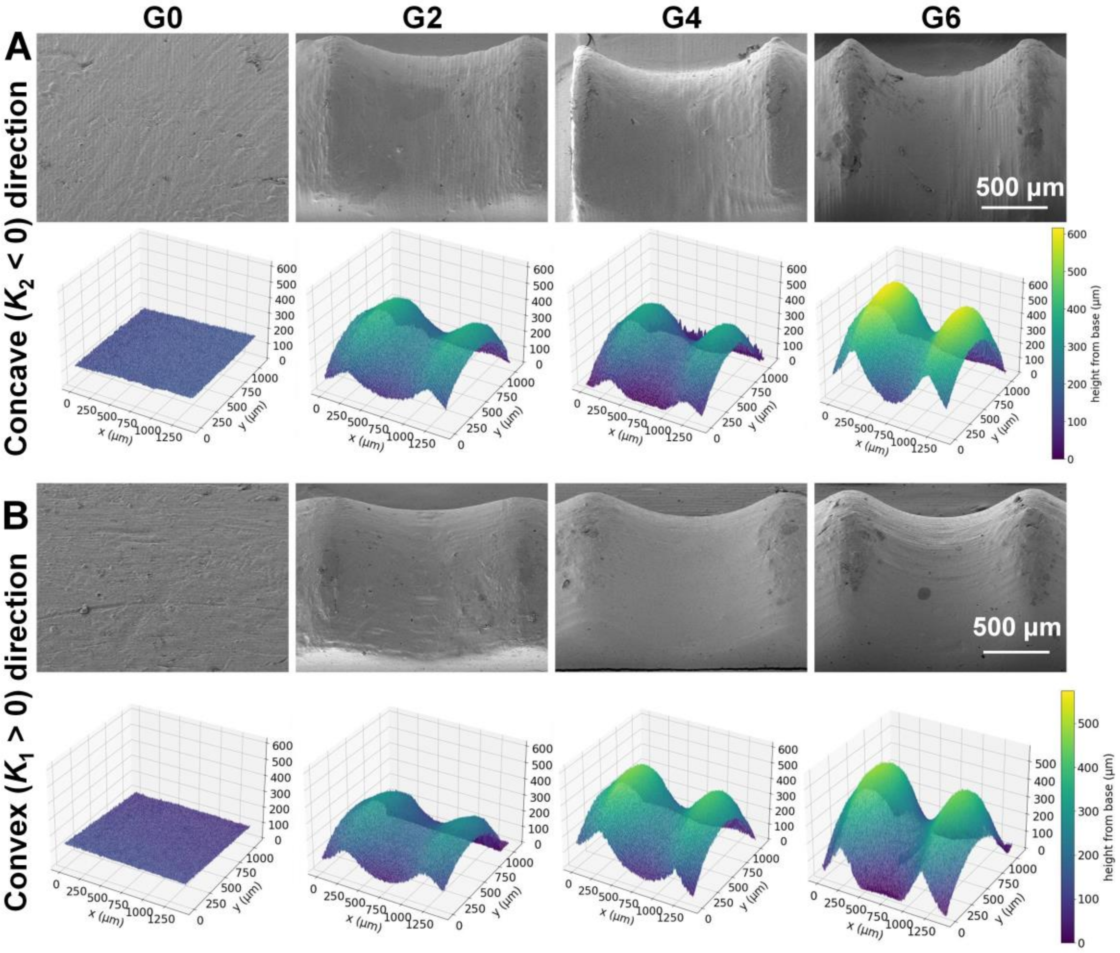

Fig. S5. Characterization of hyperboloid surface scaffolds printed in (A) the concave (K_2 < 0) direction and (B) convex (K_1 > 0) direction by SEM and 3D laser microscopy evaluation.

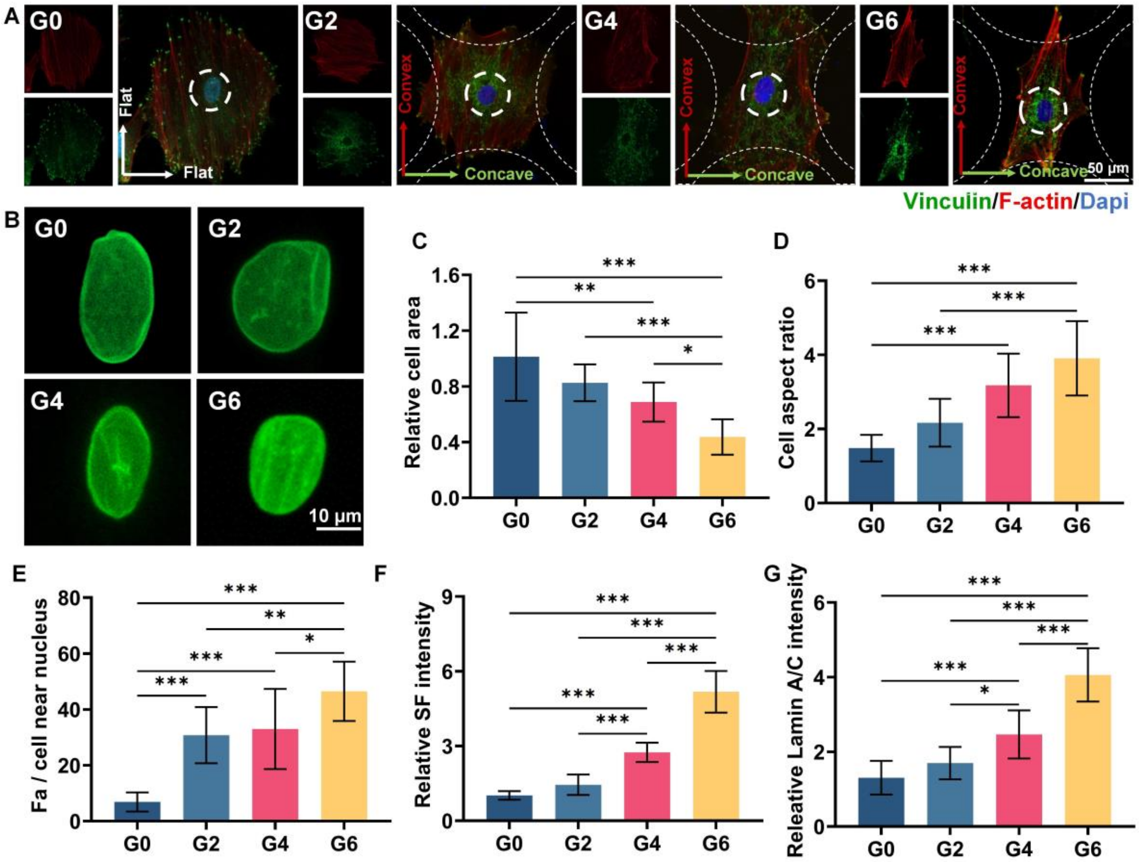

Fig. S6. Effect of hyperboloid topology printed in the convex ($K_1 > 0$) direction on stress fiber (SF), vinculin and Lamin A/C expression. (A) Immunofluorescence staining of vinculin (green), *F*-actin (red) and nuclei (blue) of hMSCs on hyperboloid surfaces; (B) Immunofluorescence staining of Lamin A/C (green) of hMSCs on hyperboloid surfaces; (C-E) Quantification of cell area, cell aspect ratio and number of focal adhesion (FA) near nuclei; (F-G) Quantification of SF and Lamin A/C (green) intensity of hMSCs on hyperboloid surfaces; Sample size $n = 10$ for all experiments by one-way ANOVA with a Tukey's post hoc test for multiple comparison. Data are presented as mean ± SD. *$p < 0.05$, **$p < 0.01$, and ***$p < 0.001$ denote the statistical significance.

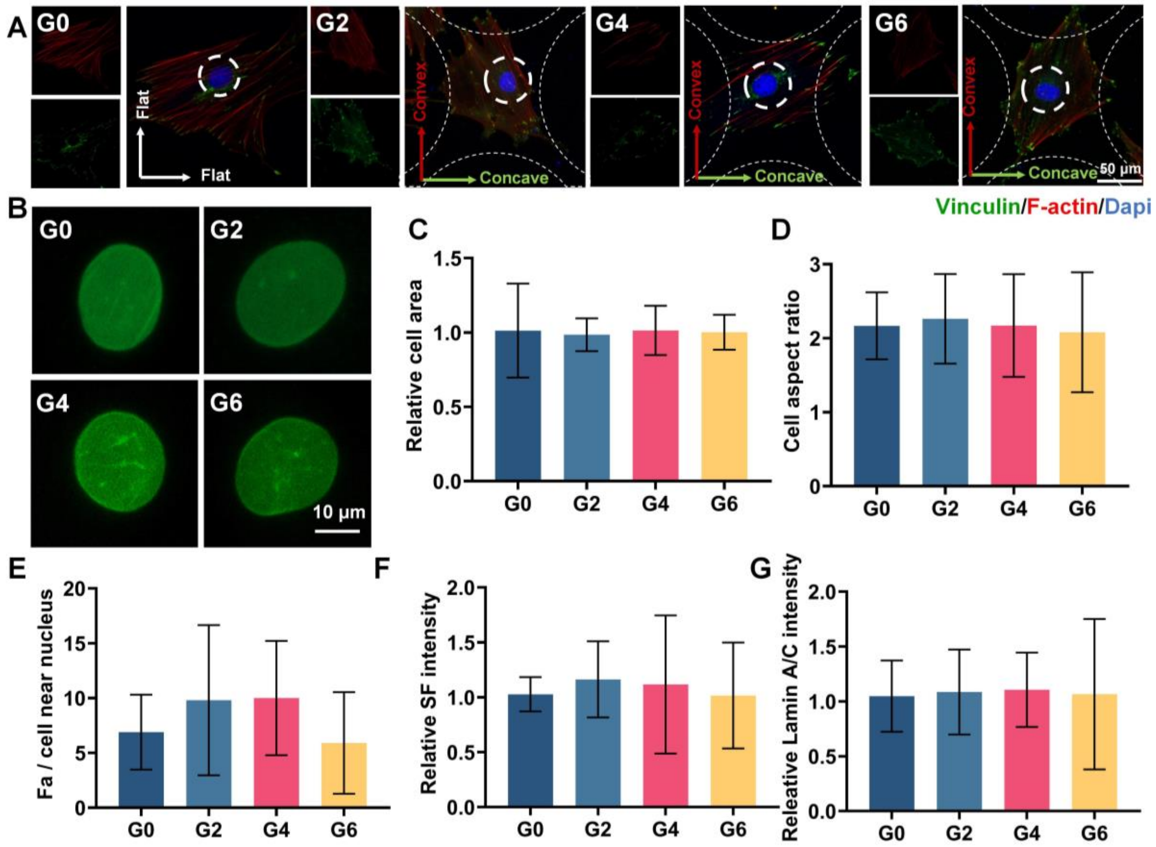

Fig. S7. Effect of focal adhesion kinase (FAK) inhibition by PF-573228 on stress fiber (SF), vinculin and Lamin A/C expression. (A) Immunofluorescence staining of vinculin (green), *F*-actin (red) and nuclei (blue) of hMSCs on hyperboloid surfaces; (B) Immunofluorescence staining of Lamin A/C (green) of hMSCs on hyperboloid surfaces; (C-E) Quantification of cell area, cell aspect ratio and number of focal adhesion (FA) near nuclei; (F-G) Quantification of SF and Lamin A/C (green) intensity of hMSCs on hyperboloid surfaces; Sample size *n* = 10 for all experiments. Data are presented as mean ± SD.

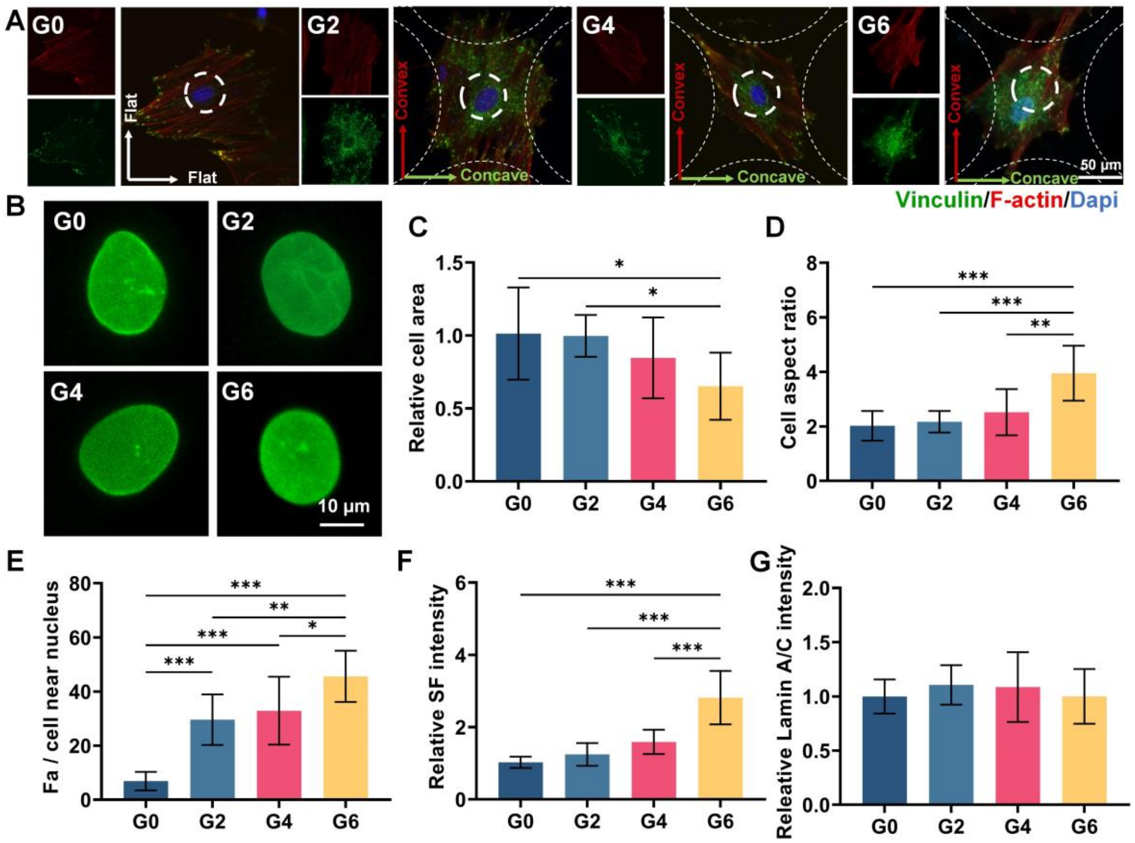

Fig. S8. Effect of myosin II inhibition by Blebbistatin on stress fiber (SF), vinculin and Lamin A/C expression. (A) Immunofluorescence staining of vinculin (green), *F*-actin (red) and nuclei (blue) of hMSCs on hyperboloid surfaces; (B) Immunofluorescence staining of Lamin A/C (green) of hMSCs on hyperboloid surfaces; (C-E) Quantification of cell area, cell aspect ratio and number of focal adhesion (FA) near nuclei; (F-G) Quantification of SF and Lamin A/C (green) intensity of hMSCs on hyperboloid surfaces; Sample size $n = 10$ for all experiments by one-way ANOVA with a Tukey's post hoc test for multiple comparison. Data are presented as mean ± SD. *$p < 0.05$, **$p < 0.01$, and ***$p < 0.001$ denote the statistical significance.

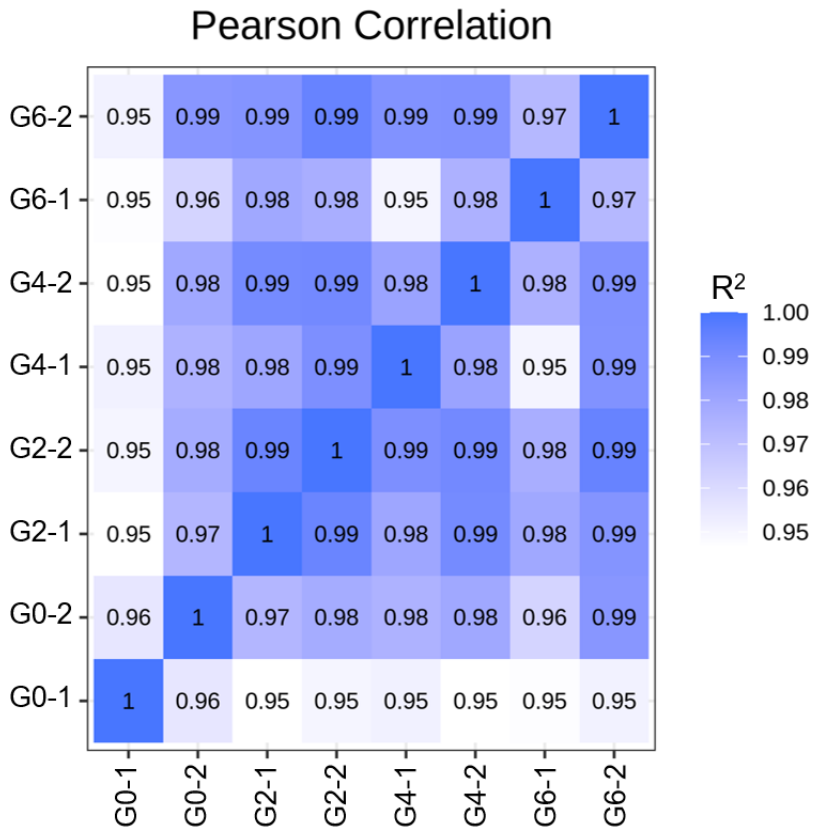

Fig. S9. Pearson correlation evaluation between different samples.

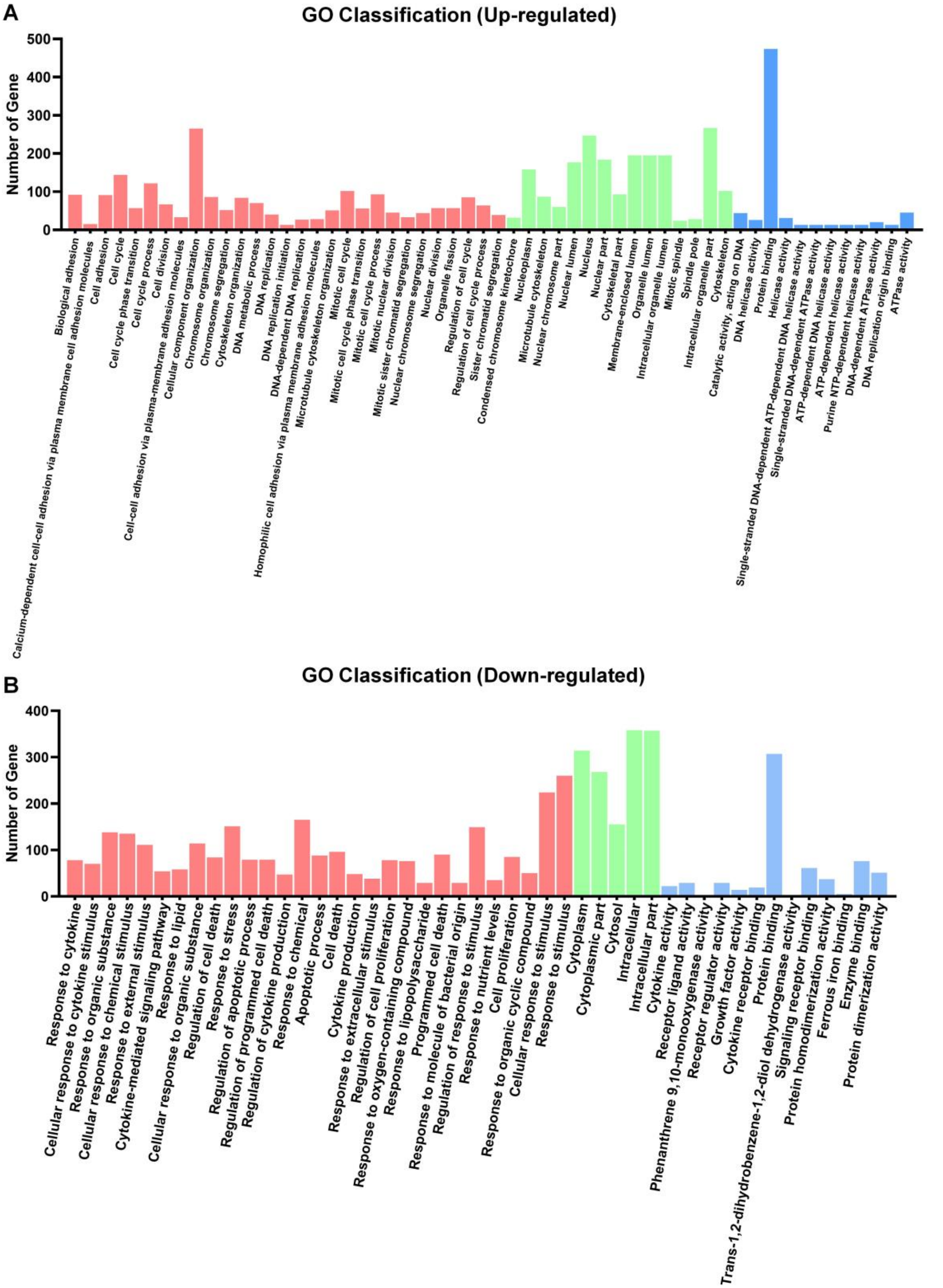

Fig. S10. GO classification of (A) up-regulated and (B) down-regulated differentially expressed genes of hMSCs (G6 versus G0).

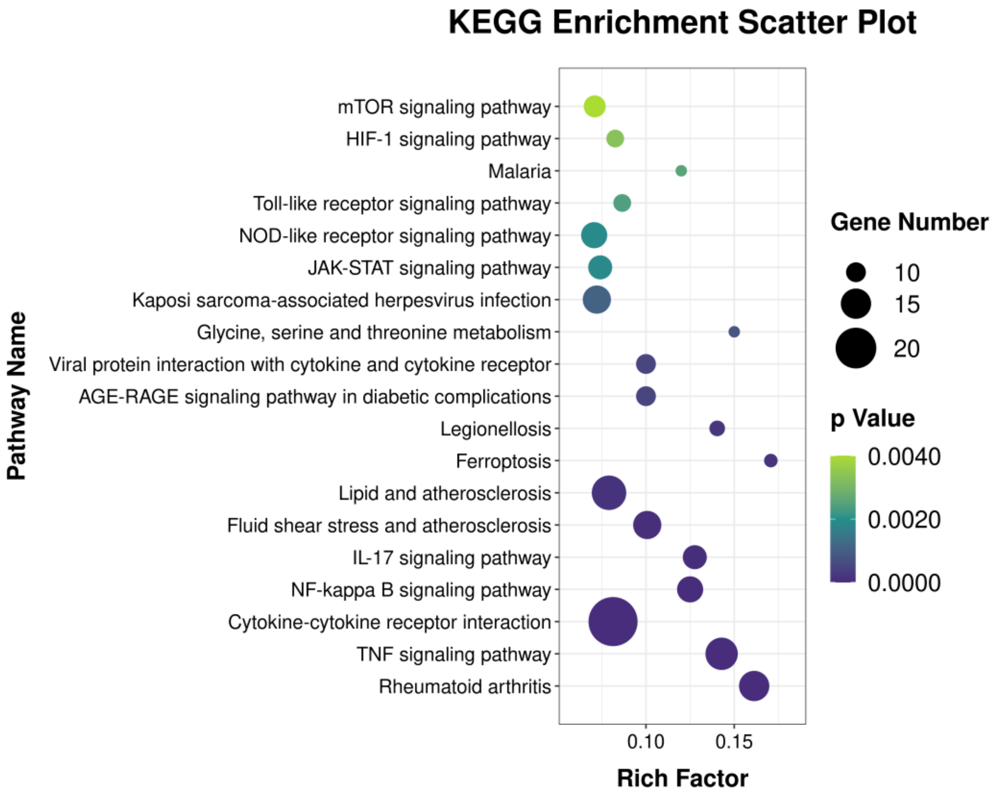

Fig. S11. Down-regulated enriched KEGG pathways of hMSCs (G6 versus G0).

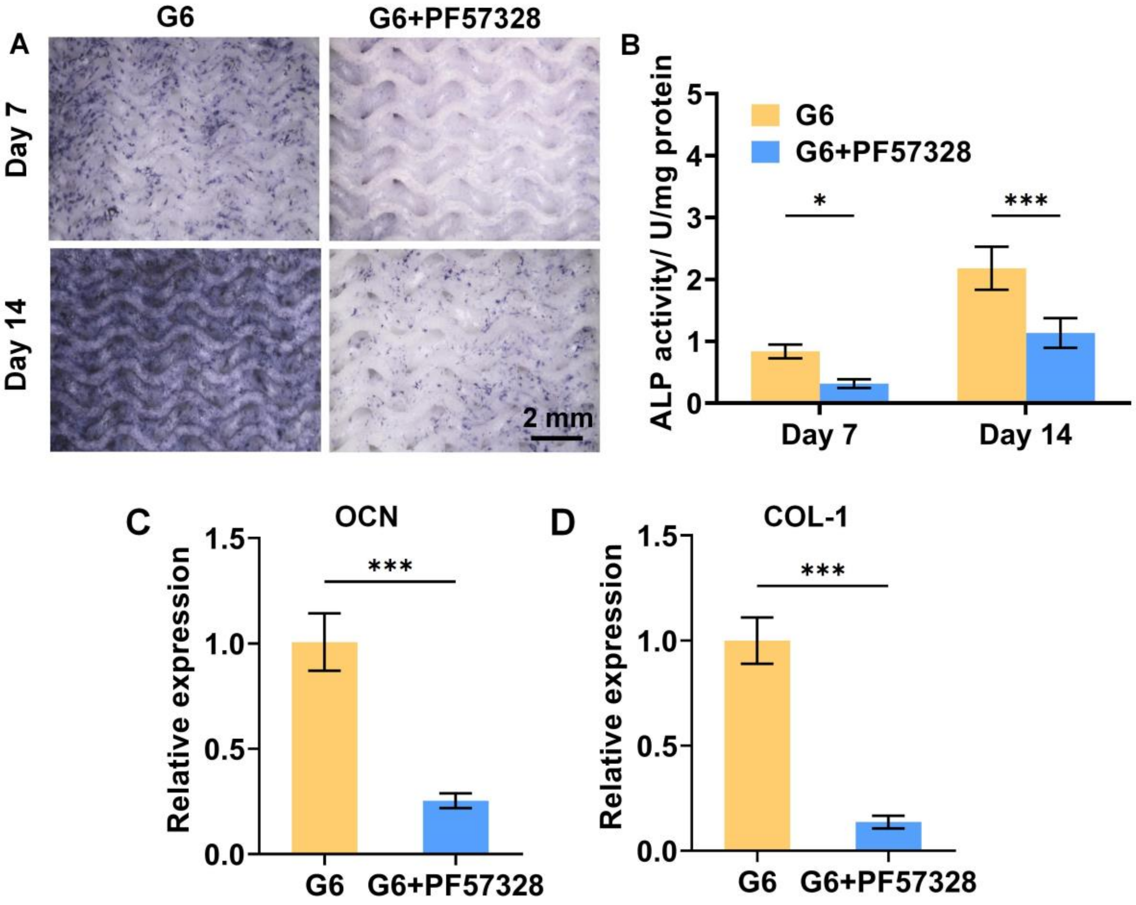

Fig. S12. Effect of focal adhesion kinase (FAK) inhibition by PF-573228 in FAK pathway on the osteogenesis of hMSCs on TPMS scaffolds. G6 group was used since it expressed the most differentially genes compared with the G0 group. (A and B) ALP staining and ALP activity; (C and D) Relative osteogenic gene expression. Data are presented as mean ± SD. *$p < 0.05$, and ***$p < 0.001$ denote the statistical significance.

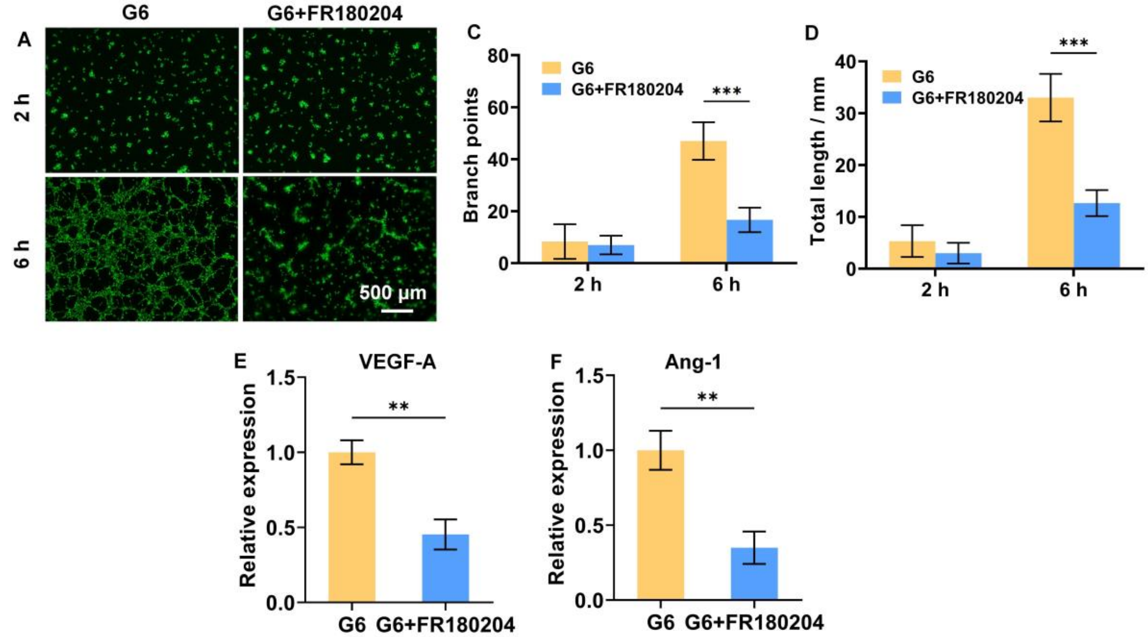

Fig. S13. Effect of ERK 1/2 inhibition by FR180204 in MAPK pathway on the angiogenic paracrine response of hMSCs on TPMS scaffolds. G6 group was used since it expressed the most differentially genes compared with the G0 group. (A to D) Tube formation assay; (E and F) Relative angiogenic gene expression of HUVECs. Data are presented as mean ± SD. $^{**}p < 0.01$, and $^{***}p < 0.001$ denote the statistical significance.

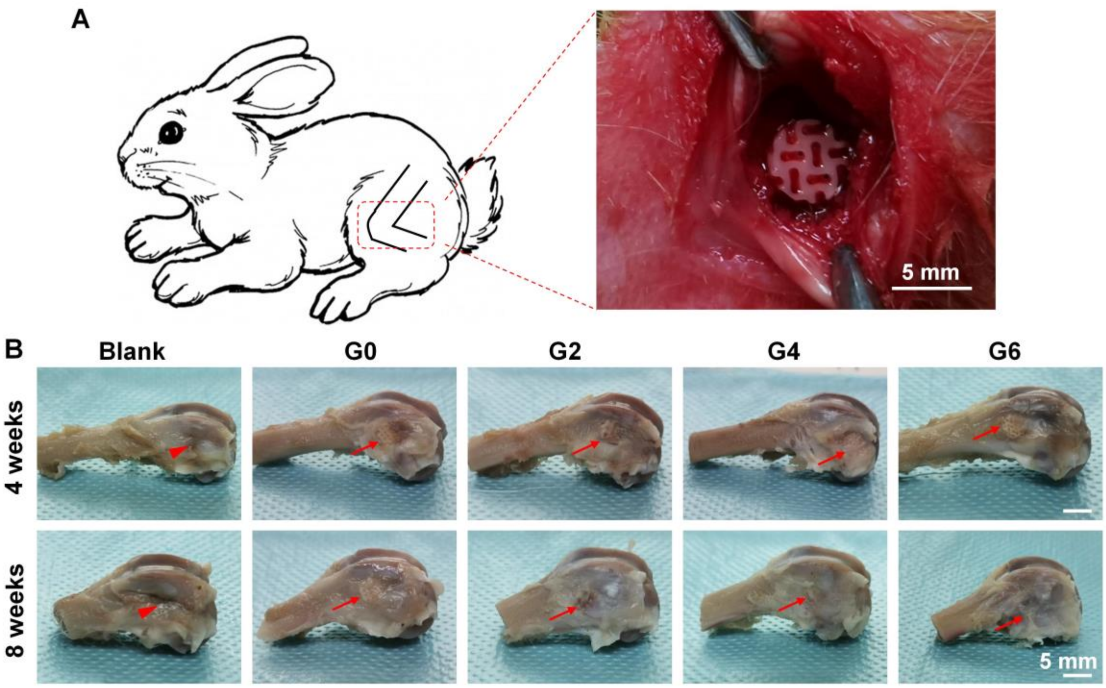

Fig. S14. Bone regeneration evaluation with a rabbit femur defect model. (A) Schematic and digital images showing the scaffold implantation process. (B) Representative macroscopic images showing the bone regeneration using the blank (no scaffold), G0 (conventional truss scaffolds), G2, G4 and G6 TPMS scaffolds after implantation of 4 and 8 weeks. The red arrow indicates the bone defect/scaffold area.

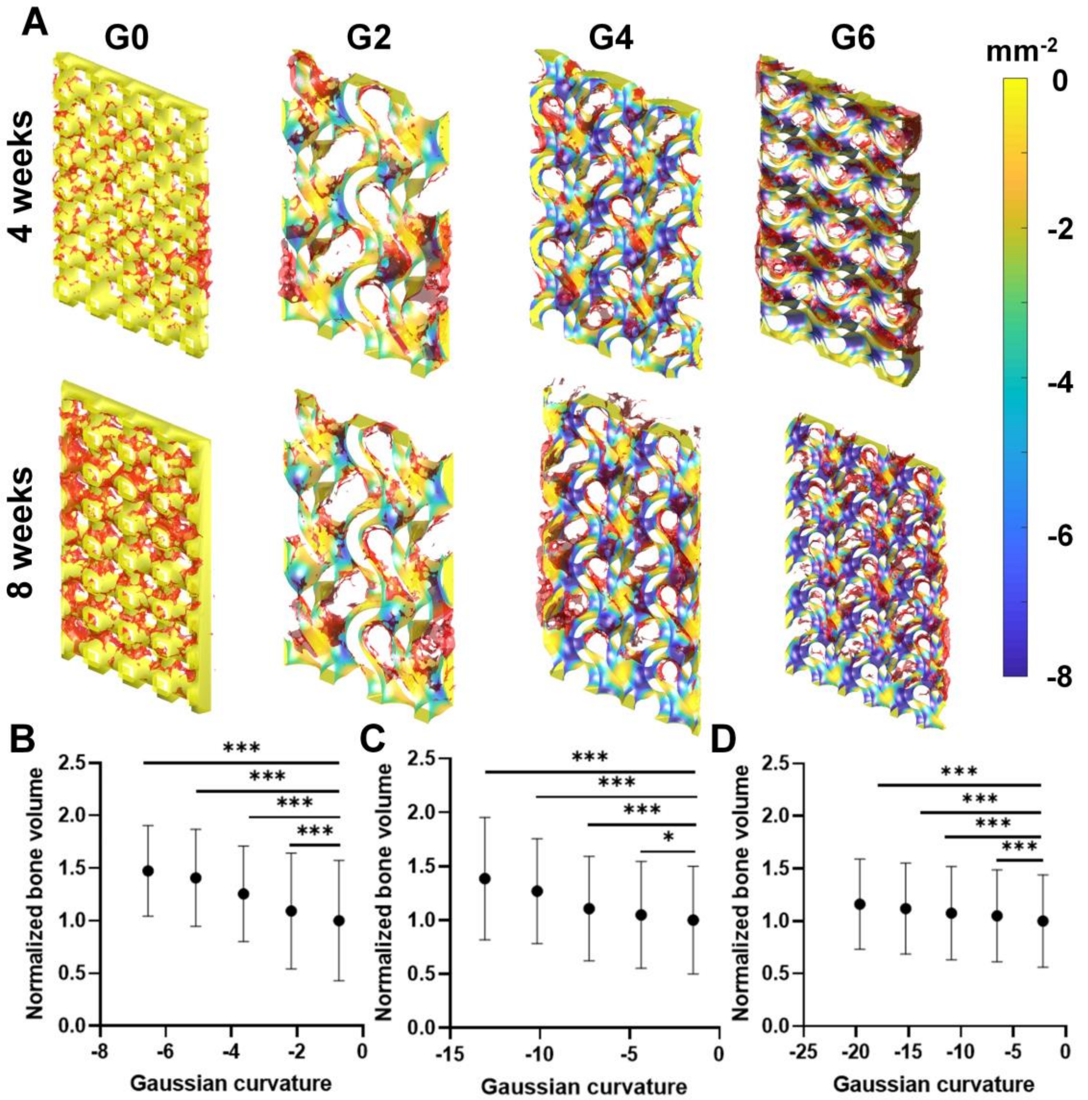

Fig. S15. (A) Spatial correlation between the local Gaussian curvature and the new bone formation in each TPMS scaffold. The red color indicates new bone formation. The blue and yellow color indicates high Gaussian curvature and low Gaussian curvature regions in each scaffold. (B-D) The quantification of the new bone formation against the local Gaussian curvature for G2 (B), G4 (C), and G6 (D) scaffolds. Data are presented as mean ± SD. *$p < 0.05$, and ***$p < 0.001$ denote the statistical significance.

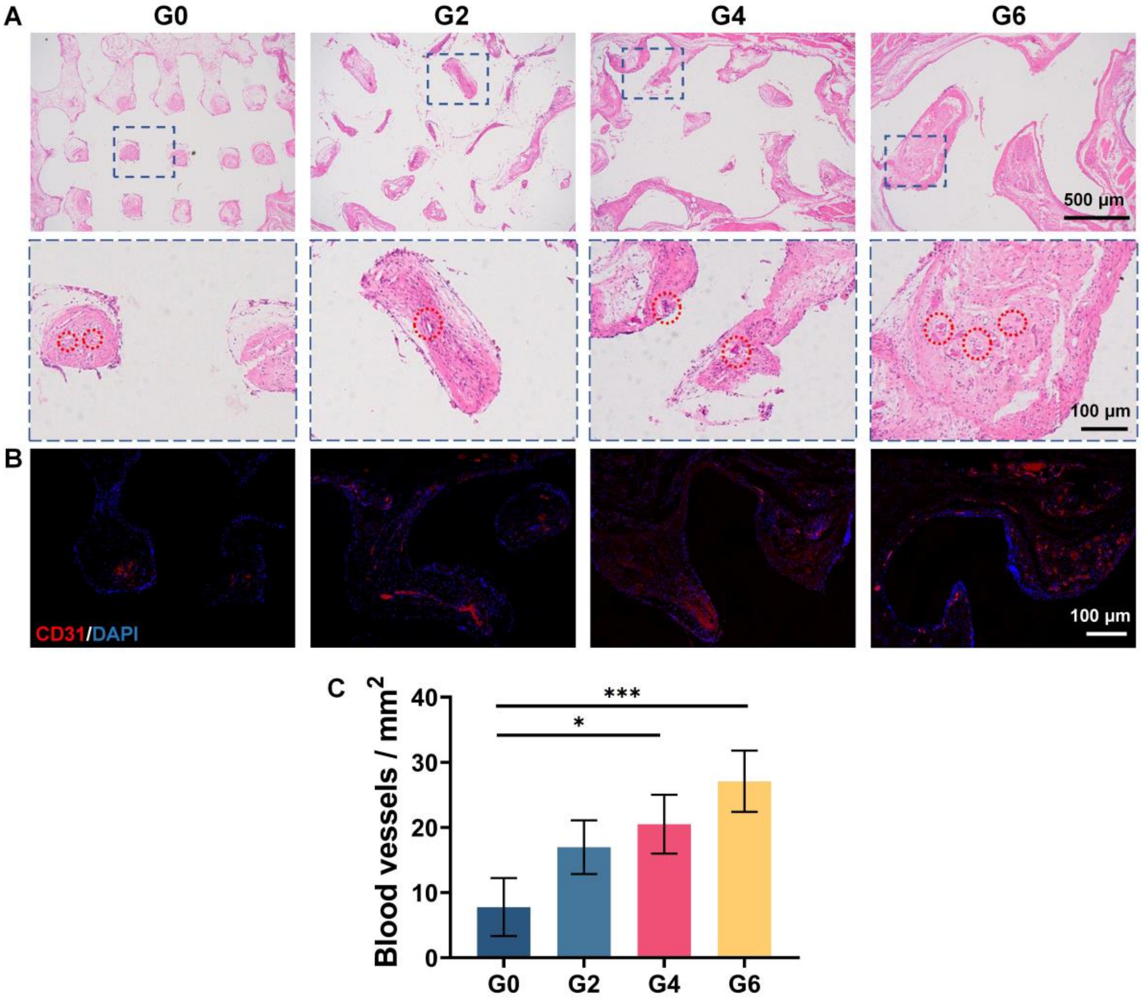

Fig. S16. Neovascularization evaluation by mouse subcutaneous implantation model. (A) H&E histological staining images of the TPMS scaffolds after 35 days of implantation. Blue box indicates the magnification sites and red circle indicates the vessels. (B) CD31 immunohistochemical staining (red) of the neovasculature. (C) Representative digital images showing the scaffold implantation process. (D) Quantification of the number of blood vessels. Sample size $n = 3$ for all experiments by a one-way ANOVA with a Tukey's post hoc test for multiple comparison. Data are presented as mean ± SD. *$p < 0.05$, and ***$p < 0.001$ denote the statistical significance.

Table S1. Reproducibility of the fabricated scaffolds determined by micro-CT

	G0	G2	G4	G6
Porosity (%)	97.78±0.79	96.67±1.36	98.89±0.79	98.89±0.79
Mean pore size (%)	95.83±1.18	94.67±2.05	97.33±0.94	97.62±2.43
Strut thickness (%)	95±2.04	95.56±1.81	93.33±2.72	95.56±4.16

$$Reproducibility\ of\ porosity = \frac{Porosity\ of\ fabricated\ scaffolds}{Porosity\ of\ designed\ scaffolds} \times 100\%$$

$$Reproducibility\ of\ mean\ pore\ size = \frac{Mean\ pore\ size\ of\ fabricated\ scaffolds}{Mean\ pore\ size\ of\ designed\ scaffolds} \times 100\%$$

$$Reproducibility\ of\ strut\ thickness = \frac{Strut\ thickness\ of\ fabricated\ scaffolds}{Strut\ thickness\ of\ designed\ scaffolds} \times 100\%$$

Table S2. Primer sequences in the qRT-PCR study

Genes	Sequences
ALP	F: 5'-AATCGGAACAACCTGACTGACCCT -3'<br>R:5'-AATCCTGCCTCCTTCCACTAGCAA-3'
OCN	F: 5'-GAGGAAGTGGGCAGGAGAATG -3'<br>R: 5'-GTAGTAGAAAGGGGACAGGAC -3'
Col-1	F: 5'-GACTTCTCCCAAGCGGGAAC-3'<br>R:5'-AGAAATTGAGCCCCAGGTTGA-3'
RUNX2	F: 5'-CAAGCACAAGTGATTGGCCGAACT-3'<br>R:5'-CCTCAACCACGAAGCCTGCAATTT-3'
GAPDH	F: 5’-TGATTCTACCCACGGCAAGTT-3’<br>R: 5’-TGATGGGTTTCCCATTGATGA-3’

Table S3. Primer sequences for genes selected for RNA-seq validation

Genes	Sequences
ITGA11	F: 5'- TCACGGACACCTTCAACATGG-3'<br>R: 5'- CCAGCCACTTATTGCCACTGA-3'
ANGPT2	F: 5'- TCACGGACACCTTCAACATGG-3'<br>R: 5'- CCAGCCACTTATTGCCACTGA-3'
MAP2K6	F: 5'- GTGGTCACCAAAGTCCAGCACA-3'<br>R: 5'- CACGATGTACGGCGAGTTGCAT-3'
IL1B	F: 5’-CCACAGACCTTCCAGGAGAATG-3’<br>R: 5’-GTGCAGTTCAGTGATCGTACAGG-3’
IL6	F: 5’-AGACAGCCACTCACCTCTTCAG-3’<br>R: 5’-TTCTGCCAGTGCCTCTTTGCTG-3’
ITGA1	F: 5'-CCGAAGAGGTACTTGTTGCAGC-3'<br>R:5'- GGCTTCCGTGAATGCCTCCTTT-3'
ITGB2	F: 5'- CAACGTATGCGAGTGCCATTC-3'<br>R:5'- TTCACGGGGTTGTTCGACAG-3'
PKT2	F: 5'- TCCTAATGTTGATGCCTGCC-3'<br>R:5'- CCTTGAAAAGGCTTCACACC-3'
VCL	F: 5’-TGAGCAAGCACAGCGGTGGATT-3’<br>R: 5’-TCGGTCACACTTGGCGAGAAGA-3’
MAPK3	F: 5’-TGGCAAGCACTACCTGGATCAG-3’<br>R: 5’-GCAGAGACTGTAGGTAGTTTCGG-3’
MAPK1	F: 5’-ACACCAACCTCTCGTACATCGG-3’<br>R: 5’-TGGCAGTAGGTCTGGTGCTCAA-3’

References:

1. H. Jinnai *et al.*, Surface curvatures of trabecular bone microarchitecture. *Bone* **30**, 191-194 (2002).
2. R. Goldman, Curvature formulas for implicit curves and surfaces. *Computer Aided Geometric Design* **22**, 632-658 (2005).
3. B. Zhang *et al.*, 3D printing of calcium phosphate bioceramic with tailored biodegradation rate for skull bone tissue reconstruction. *Bio-Design and Manufacturing* **2**, 161-171 (2019).
4. Y. Tang, M. Mao, S. Qiu, C. Wu, Annealing effects on the pore structures and mechanical properties of porous alumina via directional freeze-casting. *Journal of the European Ceramic Society* **38**, 4149-4154 (2018).
5. Q. Zhang *et al.*, High‐Strength Hydroxyapatite Scaffolds with Minimal Surface Macrostructures for Load‐Bearing Bone Regeneration. *Advanced Functional Materials*, 2204182 (2022).
6. D. Chicot *et al.*, Mechanical properties of porosity-free beta tricalcium phosphate (β-TCP) ceramic by sharp and spherical indentations. (2013).
7. A. Garcia-Prieto, J. C. Hornez, A. Leriche, P. Pena, C. Baudín, Influence of porosity on the mechanical behaviour of single phase β-TCP ceramics. *Ceramics International* **43**, 6048-6053 (2017).
8. G.-H. Yang, F. Mun, G. Kim, Direct electrospinning writing for producing 3D hybrid constructs consisting of microfibers and macro-struts for tissue engineering. *Chemical Engineering Journal* **288**, 648-658 (2016).
9. Y. Yang, T. Xu, H. P. Bei, Y. Zhao, X. Zhao, Sculpting Bio‐Inspired Surface Textures: An Adhesive Janus Periosteum. *Advanced Functional Materials*, 2104636 (2021).
10. L. Ma *et al.*, Multifunctional bioactive Nd-Ca-Si glasses for fluorescence thermometry, photothermal therapy, and burn tissue repair. *Science advances* **6** (2020).
11. Z. Yang *et al.*, Bioclickable and mussel adhesive peptide mimics for engineering vascular stent surfaces. *Proceedings of the National Academy of Sciences* **117**, 16127-16137 (2020).
12. Y. Yang *et al.*, Biomimetic, Stiff, and Adhesive Periosteum with Osteogenic–Angiogenic Coupling Effect for Bone Regeneration. *Small* **17**, 2006598 (2021).
13. Y. Yang *et al.*, Photocrosslinkable nanocomposite ink for printing strong, biodegradable and bioactive bone graft. *Biomaterials* **263**, 120378 (2020).
14. W. N. Arifin, W. M. Zahiruddin, Sample size calculation in animal studies using resource equation approach. *The Malaysian journal of medical sciences: MJMS* **24**, 101 (2017).
15. C. Yang *et al.*, 3D Printed Wesselsite Nanosheets Functionalized Scaffold Facilitates NIR‐II Photothermal Therapy and Vascularized Bone Regeneration. *Advanced Science*, 2100894 (2021).
16. M. L. Bouxsein *et al.*, Guidelines for assessment of bone microstructure in rodents using micro–computed tomography. *Journal of bone and mineral research* **25**, 1468-1486 (2010).
17. T. Wang *et al.*, Engineering immunomodulatory and osteoinductive implant surfaces via mussel adhesion-mediated ion coordination and molecular clicking. *Nature Communications* **13**, 1-17 (2022).
18. A. E. Jakus *et al.*, Hyperelastic “bone”: A highly versatile, growth factor–free, osteoregenerative, scalable, and surgically friendly biomaterial. *Science translational medicine* **8**, 358ra127-358ra127 (2016).
